# Venous thromboembolism recurrence among one-and-done direct oral anticoagulant users: a retrospective longitudinal study

**DOI:** 10.1007/s11096-023-01589-7

**Published:** 2023-05-19

**Authors:** Mark Alberts, Maryia Zhdanava, Dominic Pilon, Gabrielle Caron-Lapointe, Patrick Lefebvre, Brahim Bookhart, Akshay Kharat

**Affiliations:** 1 Hartford Hospital, Hartford, CT USA; 2 Analysis Group, Inc., Montréal, QC Canada; 3 Janssen Scientific Affairs, LLC, Titusville, NJ USA

**Keywords:** Direct oral anticoagulants, Recurrence, Treatment discontinuation, Venous thromboembolism

## Abstract

**Background:**

Direct oral anticoagulants (DOACs) are the American Society of Hematology guideline-recommended treatment for venous thromboembolism (VTE) in the United States (US).

**Aim:**

To compare risk of VTE recurrence between patients who, following the first fill, discontinued (“one-and-done”) versus those who continued (“continuers”) DOACs.

**Method:**

Open source US insurance claims data (04/1/2017 to 10/31/2020) were used to select adult patients with VTE initiated on DOACs (index date). Patients with only one DOAC claim during the 45-day landmark period (starting on the index date) were classified as one-and-done and the remaining as continuers. Inverse probability of treatment weighting was used to reweight baseline characteristics between cohorts. VTE recurrence based on the first post-index deep vein thrombosis or pulmonary embolism event was compared using weighted Kaplan–Meier and Cox proportional hazard models from landmark period end to clinical activity or data end.

**Results:**

27% of patients initiating DOACs were classified as one-and-done. After weighting, 117,186 and 116,587 patients were included in the one-and-done and continuer cohorts, respectively (mean age 60 years; 53% female; mean follow-up 15 months). After 12 months of follow-up, the probability of VTE recurrence was 3.99% and 3.36% in the one-and-done and continuer cohorts; the risk of recurrence was 19% higher in the one-and-done cohort (hazard ratio [95% confidence interval] = 1.19 [1.13, 1.25]).

**Conclusion:**

Substantial proportion of patients discontinued DOAC therapy after the first fill, which was associated with significantly higher risk of VTE recurrence. Early access to DOACs should be encouraged to reduce the risk of VTE recurrence.

**Supplementary Information:**

The online version contains supplementary material available at 10.1007/s11096-023-01589-7.

## Impact statements

- In prior research, nonadherence to direct oral anticoagulant (DOAC) therapy after ≥ 3 months of initial treatment and lack of DOAC persistence in the first 3 months was associated with an increased risk of venous thromboembolism (VTE) recurrence and hospital readmissions; however, implications of early treatment discontinuation have not been evaluated.
- In this real-world United States (US) study, patients with VTE who discontinued a DOAC after the first fill (“one-and-done”; 27%) had a 19% higher risk of VTE recurrence at 12 months of follow-up compared to patients who continued DOAC treatment.
- These findings suggest that discontinuing DOAC therapy after the first fill is common in the US, and is associated with increased risk of VTE recurrence in the short and long term.
- Development of policies and pharmacy programs to encourage or monitor use of DOACs beyond the first fill may help to protect patients from life-threatening VTE events in both the short and long terms.

## Introduction

Venous thromboembolism (VTE) manifests as a clinical event driven by dysregulated coagulation, encompassing both deep vein thrombosis (DVT) and pulmonary embolism (PE) [1]. In 2016, 1.2 million people were reported to have incident VTE in the United States (US), and the number of VTE events has been steadily increasing over the last 2 decades [1].

VTE can be triggered by several risk factors, including active cancer, surgery, prolonged immobilization, major trauma, infection, and oral contraception [2, 3]. Timely diagnosis of VTE is important, since if left untreated, VTE can result in cardiopulmonary collapse, long-term complications, and death [2]. Additionally, VTE recurrence is common, with a 10-year recurrence rate of 30% [1]. Notably, active cancer and unprovoked initial VTE increase the risk of VTE recurrence, while persistent anticoagulation treatment can significantly reduce the risk [4–6].

Treatment of VTE involves the use of anticoagulation, mainly direct oral anticoagulants (DOACs) [7]. In the US, a portion of the cost of DOACs may be covered by insurance (e.g., commercial plan, Medicare, Medicaid) and a portion is paid out-of-pocket by the patient. There are currently 4 DOACs approved by the US Food and Drug Administration for VTE, including rivaroxaban, apixaban, dabigatran, and edoxaban [8–11]. In clinical trials, DOACs demonstrated similar reductions in the risk of recurrence and death as vitamin K antagonists but were associated with a significantly decreased risk of major bleeding (combined relative risk [95% confidence interval (CI)] = 0.61 [0.45, 0.83]) [12], making DOACs the treatment of choice for the initial management of VTE [7].

The effectiveness of DOACs is contingent on patient adherence and persistence with treatment. Previous research reported an association between nonadherence to DOAC therapy and an increased risk of VTE recurrence among patients with VTE and > 90 days of initial treatment [5]. Similarly, adherence to extended DOAC therapy after the 6 months of initial treatment was associated with lower risk of VTE recurrence compared to no extended treatment [4]. DOAC non-persistence during the first 90 days after VTE was also associated with increased risk of hospital or emergency room readmission [13]. Yet, implications of early DOAC discontinuation for the risk of VTE recurrence have not been evaluated.

### Aim

Therefore, this study describes and compares the risk of VTE recurrence among patients who discontinued DOAC therapy following the first fill (“one-and-done”) compared to patients who continued DOACs beyond the first fill.

### Ethics approval

Data were de-identified and comply with the patient requirements of the Health Insurance Portability and Accountability Act (HIPAA) of 1996; therefore, no review by an institutional review board was required per Title 45 of CFR, Part 46.101(b)(4) [14].

## Method

### Data source

Data (04/01/2017- 10/31/2020) from Symphony Health, an ICON plc Company, PatientSource® were used. This open claims US database contains patient demographics, medical and prescription drug claims (with status of prescription drug claims; i.e., approved, rejected, abandoned). It captures > 75% of all US retail prescription claims, representing over three-quarters of the US patients annually across multiple payer channels (i.e., commercial, Medicare, Medicaid). The open claims nature means that a patient’s healthcare activity is captured regardless of maintaining the same healthcare plan if the patient uses providers from the network that supplies data to the database.

### Study design

A retrospective longitudinal study design was used (Supplementary Fig. 1). The index date was the date of the first observed DOAC (i.e., apixaban, dabigatran, rivaroxaban) claim; only patients with the status of the first claim being “approved” (i.e., submitted by a pharmacy and approved for payment by health plans after claims adjudication) were included.

The baseline period comprised the 6 months before the index date with clinical activity (defined as either a pharmacy or medical claim; based on the first and last patient claim).

The 45-day landmark period following the index date was used to classify patients into mutually exclusive “one-and-done” (i.e., DOAC discontinuation after the index claim) and “continuer” (i.e., persistence on DOACs beyond the first claim) cohorts. The duration of the landmark period was based on a ≥ 15-day gap in DOAC supply to define DOAC discontinuation after ≤ 30 days of the index DOAC claim supply (see *Sample selection*). The gap of ≥ 15 days was chosen since 1) even brief periods of discontinuation may rapidly result in subtherapeutic anticoagulant levels [13] and 2) a shorter gap produced a shorter landmark period, minimizing the selection bias as patients with VTE recurrence during the period had to be excluded. A sensitivity analysis was conducted using the gap of ≥ 30 days resulting in a 60-day landmark period (see *Sensitivity analysis*).

The follow-up period used to measure the outcomes started on day 46 post-index and continued until the earliest of the end of clinical activity or data availability.

### Sample selection

Patients meeting the following inclusion criteria were included: (1) ≥ 1 medical claim with a diagnosis code for VTE (International Classification of Diseases, 10th Revision, Clinical Modification [ICD-10-CM]: I26.x, I80.1, I80.2, I80.3, I82.4, I82.6, I82.A1, I82.B1, I82.C1, I82.90) in any care setting, where the first observed diagnosis was defined as the index VTE; (2) the first claim for apixaban, dabigatran, or rivaroxaban occurred within 28 days after the index VTE diagnosis and had an “approved” status; (3) ≥ 6 months of clinical activity before the index date; (4) no claims for other oral anticoagulants (i.e., betrixaban, edoxaban, warfarin) any time before the index date (e.g., beyond the 6-month baseline period if data were available); and (5) ≥ 18 years old on the index date.

The following exclusion criteria were applied: (1) ≥ 1 claim with a diagnosis of atrial fibrillation (Supplementary Table 1) any time prior to or on the index date; (2) organ or tissue transplant during the baseline period; (3) pregnancy during or after the baseline period; (4) > 1 DOAC (i.e., apixaban, betrixaban, dabigatran, edoxaban, rivaroxaban) claimed on the index date; (5) > 1 final claim status (e.g., approved and abandoned) for the index DOAC on the index date; and (6) ≥ 1 claim with a VTE diagnosis in an inpatient setting on the index date.

Additionally, to identify the cohorts, the following exclusion criteria were applied during the landmark period: (1) ≤ 45 days of clinical activity post-index; (2) recurrent VTE diagnosis in an inpatient setting during the landmark period; and (3) > 30 days of supply on the index DOAC claim.

One-and done cohort consisted of patients who discontinued DOAC therapy after the index DOAC claim, i.e. had no approved DOAC claims between the last day of supply of the index DOAC claim and the end of the landmark period (i.e., ≥ 15-day gap in DOAC supply). Continuer cohort included patients who persisted on DOAC therapy beyond the index DOAC claim, i.e. had ≥ 2 approved DOAC claims during the landmark period.

### Outcome measures

The outcome of interest was VTE recurrence, which was measured in the one-and-done and continuer cohorts separately. VTE recurrence was defined as the first DVT or PE event (Supplementary Table 1) occurring in an inpatient setting during the follow-up period, reported as a composite outcome (i.e., DVT/PE) and separately.

### Statistical analysis

Baseline characteristics were balanced between cohorts using inverse probability of treatment weighting (IPTW). The propensity score was computed from a logistic regression model adjusting for all demographic covariates and clinical covariates that were unbalanced: age; sex; region of residence; insurance plan type; index year; Quan-Charlson Comorbidity Index (Quan-CCI) [16]; time from most recent ischemic stroke to index date; time from most recent hemorrhagic stroke to index date; number of unique prescription drugs used; use of antihypertensive agents, antihyperlipidemic agents, or antiplatelet agents; index DOAC medication; diagnosis of other serious infections (Supplementary Table 1); use of oral contraceptive pills; number of emergency department visits; and number of inpatient days. Standardized difference was used to assess the balance of baseline characteristics (< 10% indicated balance) [17]. Continuous variables were described using means, standard deviations (SDs), and medians; categorical variables were described using frequencies and proportions.

Weighted Kaplan Meier survival analysis was used to estimate the probability of the first VTE recurrent event in each cohort during the follow-up period. Comparison of the risk of recurrence between cohorts was conducted using weighted Cox proportional hazard models, with hazard ratios (HRs) and their 95% CIs and *p*-values reported. Time to the first recurrent event was measured from day 46 post-index (i.e., first day after the landmark period); patients without an observed event during the follow-up period were censored at the end of the follow-up period.

### Sensitivity analysis

A sensitivity analysis using a 60-day (vs. 45-day) landmark period was conducted to evaluate the tradeoff between the loss of patients with outcomes during days 45–60 and a potentially higher risk of VTE recurrence due to a longer time off DOAC treatment (i.e., ≥ 30 vs. ≥ 15 days) in the one-and-done cohort.

## Results

**Fig. 1:**
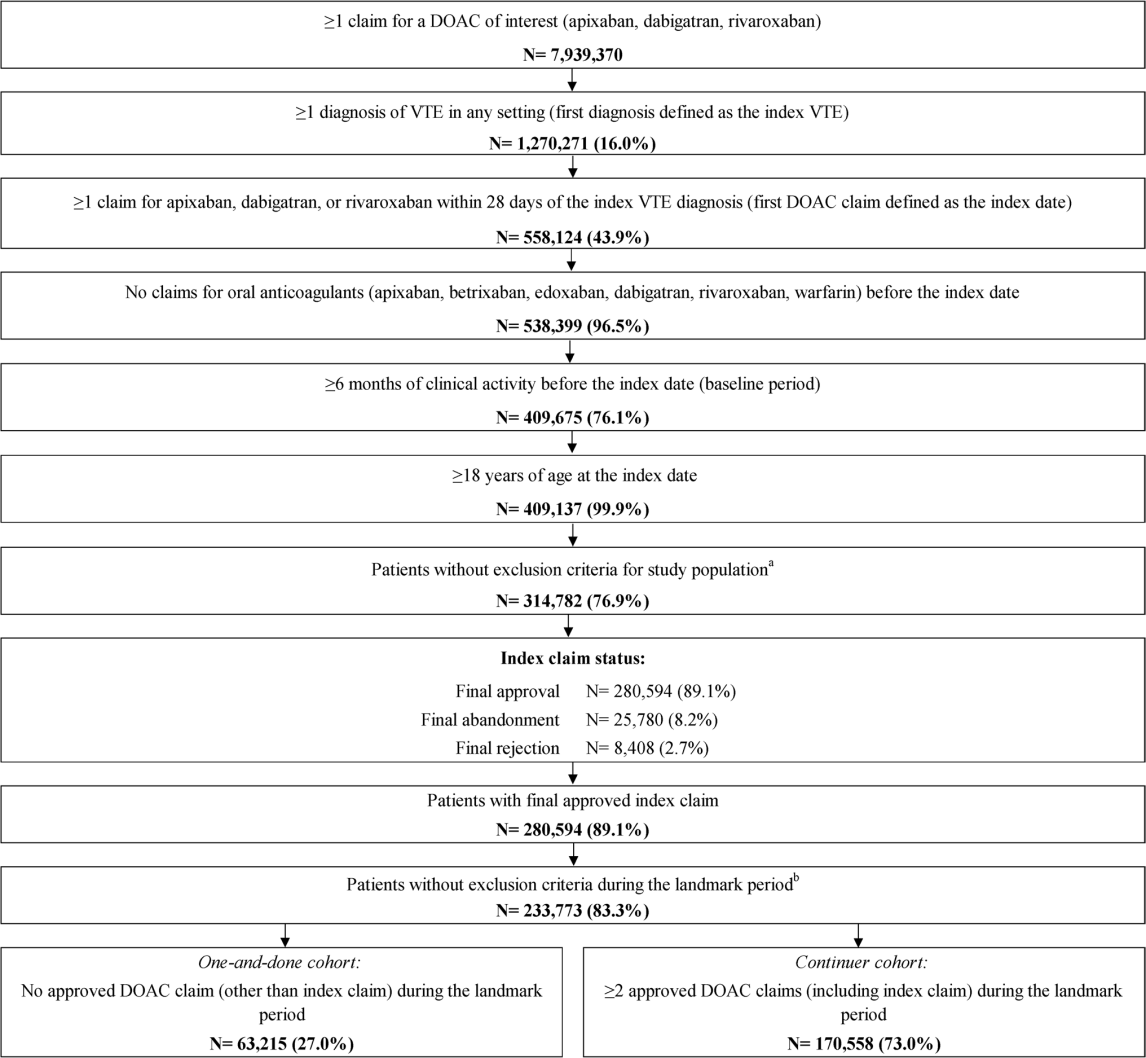
Identification of VTE population. *DOAC* Direct oral anticoagulant; *VTE* Venous thromboembolism. *Notes*: (a) Patients were excluded from the study population if they had (1) ≥ 1 claim with a diagnosis of atrial fibrillation any time prior to or on the index date; (2) organ or tissue transplant during the baseline period; (3) pregnancy during or after the baseline period; (4) > 1 DOAC (i.e., apixaban, betrixaban, dabigatran, edoxaban, rivaroxaban) claimed on the index date; (5) > 1 final claim status (e.g., approved and abandoned) for the index DOAC on the index date; and (6) ≥ 1 claim with a diagnosis of VTE in an inpatient setting on the index date. (b) Patients were excluded during the landmark period if they had (1) ≤ 45 days of clinical activity post-index; (2) a recurrent VTE diagnosis in an inpatient setting within the first 45 days post-index (i.e., during landmark period); or (3) > 30 days of supply on the index DOAC claim

Among 314,782 identified patients with VTE who were prescribed a DOAC, 280,594 (89.1%) had their first DOAC claim approved. Additional criteria during the landmark period were met by 233,773 patients, of which 63,215 (27.0%) were classified into the one-and-done cohort and 170,558 (73.0%) into the continuer cohort (Fig. 1).

### Study population and weighted baseline characteristics

After weighting, a total of 117,186 patients comprised the one-and-done cohort and 116,587 patients comprised the continuer cohort. Patient baseline characteristics were balanced between the weighted cohorts (Table 1). Mean age was 59.6 years in the one-and-done cohort and 59.7 years in the continuer cohort; 53.1% and 53.0% of patients were female, respectively. Half of patients were covered by commercial insurance (49.5% in the one-and-done cohort and 50.0% in the continuer cohort), followed by Medicare (35.4% and 35.1%), Medicaid (13.9% and 13.6%), and another type of insurance (1.3% and 1.3%). Most patients had DVT as their index VTE diagnosis (58.7% in the one-and-done cohort and 54.0% in the continuer cohort), followed by PE (32.5% and 34.6%), with the remaining patients having both DVT and PE (8.8% and 11.5%). The mean time from the index VTE diagnosis to the index date was 4.7 days in the one-and-done cohort and 4.6 days in the continuer cohort. The mean patient out-of-pocket paid amount for the index drug claim was $70.30 in the one-and-done cohort and $60.70 in the continuer cohort. Patients in the one-and-done and continuer cohorts had similar prevalence rates of risk factors for VTE, with the most common being hypertension (46.9% and 43.1%, respectively), hyperlipidemia (29.3% and 28.1%), and diabetes (20.7% and 17.1%).

**Table 1:** Selected baseline characteristics in the weighted cohorts^a^

Mean ± SD [median] or n (%)	One-and-done cohort	Continuer cohort	Standardized difference (%)
	N = 117,186	N = 116,587	
Age (years)	59.6 ± 15.3 [62.0]	59.7 ± 14.7 [62.0]	0.1
Female	62,281 (53.1)	61,818 (53.0)	0.2
Region of residence			
South	48,457 (41.4)	48,117 (41.3)	0.2
Midwest	28,056 (23.9)	28,001 (24.0)	0.2
Northeast	21,790 (18.6)	21,652 (18.6)	0.1
West	18,579 (15.9)	18,512 (15.9)	0.1
Unknown	305 (0.3)	304 (0.3)	0.0
Insurance plan			
Commercial	57,967 (49.5)	58,291 (50.0)	1.1
Medicare	41,476 (35.4)	40,871 (35.1)	0.7
Medicaid	16,231 (13.9)	15,898 (13.6)	0.6
Other	1512 (1.3)	1528 (1.3)	0.2
Year of index date			
2017	6763 (5.8)	6648 (5.7)	0.3
2018	39,999 (34.1)	39,801 (34.1)	0.0
2019	42,605 (36.4)	42,465 (36.4)	0.1
2020	27,820 (23.7)	27,672 (23.7)	0.0
Patient out-of-pocket paid amount for the index drug claim (US $2021)	70.3 ± 166.0 [10.5]	60.7 ± 143.7 [10.5]	6.2
Quan-Charlson Comorbidity Index	1.7 ± 2.3 [1.0]	1.6 ± 2.3 [1.0]	1.1
RIETE score for risk of major bleeding^b^	1.30 ± 1.35 [1.00]	1.28 ± 1.32 [1.00]	1.1
Type of index VTE diagnosis^*c*^			
DVT	68,804 (58.7)	62,907 (54.0)	9.6
PE	38,093 (32.5)	40,318 (34.6)	4.4
Both DVT and PE	10,289 (8.8)	13,362 (11.5)	8.9
Baseline diagnosis for stroke in an inpatient setting^*c*^			
Ischemic stroke	1149 (1.0)	1111 (1.0)	0.3
Time from the most recent ischemic stroke to index date (days)^d^	32.7 ± 45.6 [12.0]	29.8 ± 40.9 [13.0]	6.7
Hemorrhagic stroke	297 (0.3)	288 (0.2)	0.1
Time from the most recent hemorrhagic stroke to index date (days)^d^	42.5 ± 49.5 [21.0]	38.8 ± 44.5 [20.0]	7.8
Major bleeding	1806 (1.5)	1575 (1.4)	1.6
Number of unique prescription drugs used	7.9 ± 6.8 [7.0]	7.7 ± 6.1 [6.0]	3.5
Polypharmacy (use of ≥ 5 different medications concurrently)	59,152 (50.5)	59,630 (51.1)	1.3
Use of non-oral anticoagulants	15,531 (13.3)	17,424 (14.9)	4.9
Use of cardiovascular-related medications	58,189 (49.7)	58,527 (50.2)	1.1
Antihypertensive agents	47,666 (40.7)	46,512 (39.9)	1.6
Antihyperlipidemic agents	35,047 (29.9)	34,069 (29.2)	1.5
Antiplatelet agents	5342 (4.6)	5183 (4.4)	0.5
Cancer diagnoses and/or treatments^c^	20,692 (17.7)	22,497 (19.3)	4.2
Index DOAC medication			
Apixaban	69,484 (59.3)	69,664 (59.8)	0.9
Rivaroxaban	47,001 (40.1)	46,231 (39.7)	0.9
Dabigatran	702 (0.6)	692 (0.6)	0.1
Prevalent risk factors for VTE^*c*^			
Hypertension	54,956 (46.9)	50,242 (43.1)	7.6
Hyperlipidemia	34,318 (29.3)	32,719 (28.1)	2.7
Diabetes	24,303 (20.7)	19,958 (17.1)	9.3
Obesity	17,548 (15.0)	17,616 (15.1)	0.4
Other serious infections^e^	17,026 (14.5)	16,602 (14.2)	0.8
Contraceptive pill (use of oral)	4706 (4.0)	4772 (4.1)	0.4
All-cause monthly resource utilization			
Number of days of inpatient admission	0.35 ± 0.83 [0.00]	0.34 ± 0.85 [0.00]	1.2
Number of emergency department visits	0.11 ± 0.21 [0.00]	0.10 ± 0.21 [0.00]	1.9
Number of outpatient visits	0.91 ± 1.62 [0.50]	0.87 ± 1.46 [0.50]	2.4
All-cause monthly pharmacy costs and medical charges (US $2021)^f^	4380 ± 12,987 [1,179]	4151 ± 11,349 [1,160]	1.9

*DOAC* Direct oral anticoagulant; *DVT* Deep-vein thrombosis; *PE* Pulmonary embolism; *RIETE* Registro Informatizado de la Enfermedad TromboEmbolica Venosa; *SD* Standard deviation; *US* United States; *VTE* Venous thromboembolism

^a^Cohorts were weighted on baseline characteristics using inverse probability of treatment weights; characteristics were considered well balanced if standardized difference is < 10%

^b^RIETE score for risk of major bleeding as described by Ruiz-Gimenez et al. [15]

^c^Diagnosis codes are listed in Supplementary Table 1. Drug and procedure codes for cancer treatments are listed in Supplementary Table 2 and Supplementary Table 3

^d^Among patients that have a diagnosis for the event of interest during the baseline period

^e^Other serious infections comprise: intestinal infectious diseases; tuberculosis; bacterial diseases; infections with a predominantly sexual mode of transmission; other spirochetal diseases; rickettsioses; viral and prion infections of the central nervous system; arthropod-borne viral fevers and viral hemorrhagic fevers; viral infections characterized by skin and mucous membrane lesions; other human herpesviruses; viral hepatitis; human immunodeficiency virus disease; other viral diseases; mycoses; protozoal diseases; helminthiases; pediculosis, acariasis and other infestations; sequelae of infectious and parasitic diseases; bacterial and viral infectious agents; and other infectious diseases

^f^Medical costs are reported as charged amounts (i.e., payment amount for the entire claim as requested by provider); pharmacy costs are reported from a payer’s perspective and comprise the plan paid amounts and patient copay amounts

### Composite outcome of VTE recurrence

**Fig. 2:**
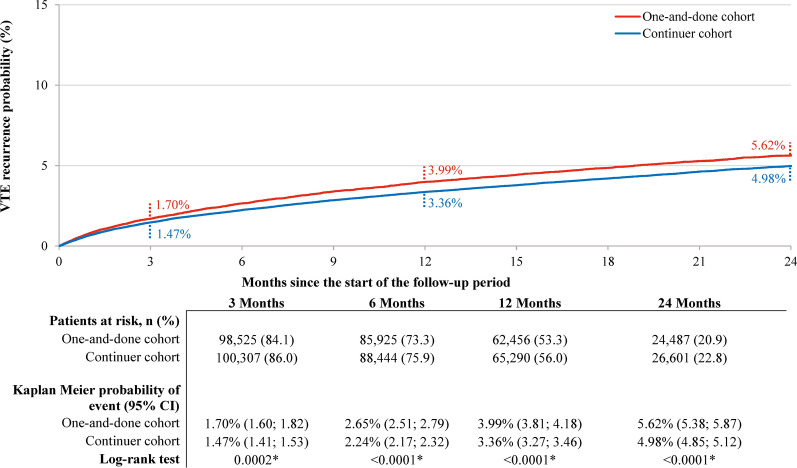
VTE recurrence (DVT or PE) probability in weighted one-and-done versus continuer cohorts.^a^ *CI* Confidence interval; *DOAC* Direct oral anticoagulant; *DVT* Deep-vein thrombosis; *PE* Pulmonary embolism; *VTE* Venous thromboembolism. *Note:* (a) VTE recurrence was identified in an inpatient setting. The time to the first recurrent event was measured from day 46 post-index date (i.e., first day after the end of the landmark period); patients for whom the event was not observed during the follow-up period were censored at the end of the follow-up period

The mean [SD] duration of follow-up was 14.7 [10.0] months in the one-and-done cohort and 15.3 [9.9] months in the continuer cohort. At 3 months of follow-up, the probability of VTE recurrence as a composite outcome of DVT or PE was higher in the one-and-done cohort (1.70%) compared to the continuer cohort (1.47%; log-rank *p*-value = 0.0002; Fig. 2). This difference was sustained at later points of follow-up, with probabilities of 3.99% in the one-and-done cohort and 3.36% in the continuer cohort at 12 months (log-rank *p*-value < 0.0001).

**Fig. 3:**
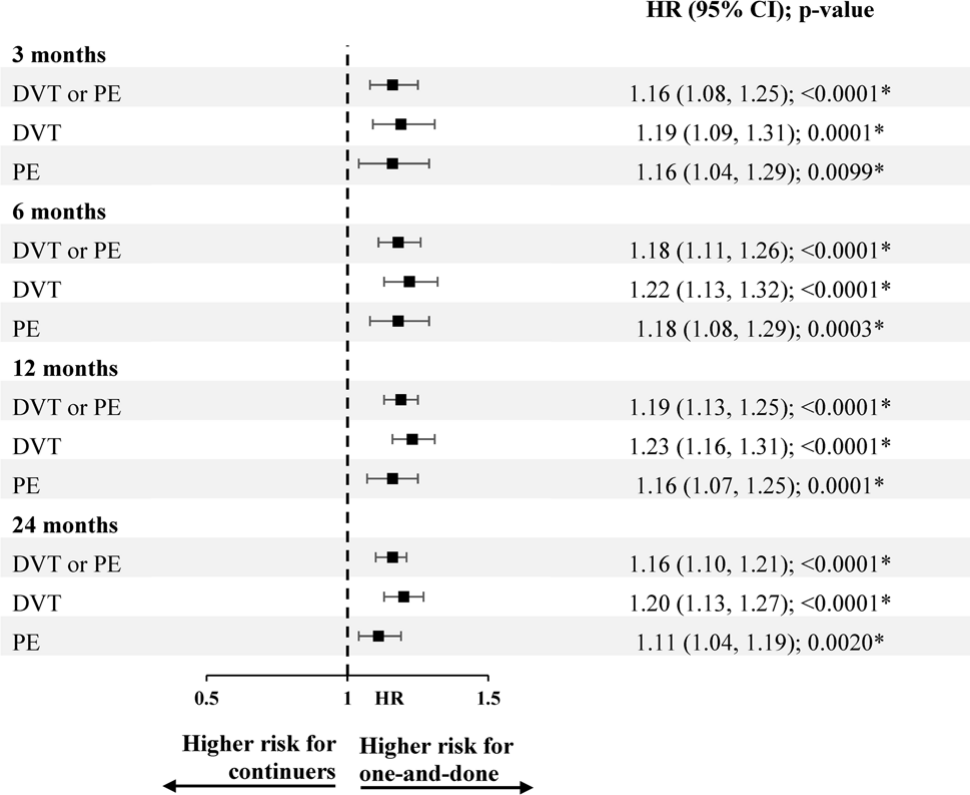
Risk of VTE recurrence in weighted one-and-done versus continuer cohorts.^a^ *p-value < 0.05; *CI* Confidence interval; *DVT* Deep-vein thrombosis; *HR* Hazard ratio; *PE* Pulmonary embolism; *VTE* Venous thromboembolism. *Note*: (a) VTE recurrence was identified in an inpatient setting. HRs were generated using univariate weighted Cox proportional hazard models

Based on weighted Cox proportional hazard analyses, there was a 16% higher risk of VTE recurrence in the one-and-done cohort compared to the continuer cohort at 3 months, 18% higher risk at 6 months, 19% higher risk at 12 months, and 16% higher risk at 24 months of follow-up (all *p*-values < 0.0001; Fig. 3).

### VTE recurrence, DVT and PE separately

The probability of DVT alone was 1.13% in the one-and-done cohort compared to 0.95% in the continuer cohort at 3 months of follow-up (log-rank *p*-value = 0.0002) and 2.67% compared to 2.17%, respectively, at 12 months (log-rank *p*-value < 0.0001; data not shown). The probability of PE alone was 0.80% in the one-and-done cohort compared to 0.69% in the continuer cohort at 3 months of follow-up (log-rank *p*-value = 0.0115) and 1.95% compared to 1.69%, respectively, at 12 months (log-rank *p*-value = 0.0002; data not shown). As DVT is more frequently treated in outpatient settings than PE [18], DVT recurrence may have been underestimated in this analysis since recurrence was only identified in the inpatient setting.

There was a 19–23% higher risk of DVT alone in the one-and-done cohort compared to the continuer cohort at 3, 6, 12, and 24 months of follow-up (all *p*-values ≤ 0.0001). There was a 11–18% higher risk of PE alone in the one-and-done cohort compared to the continuer cohort at 3, 6, 12, and 24 months of follow-up (*p*-values = 0.0001–0.0099; Fig. 3).

### Sensitivity analysis using a 60-day landmark period

**Fig. 4:**
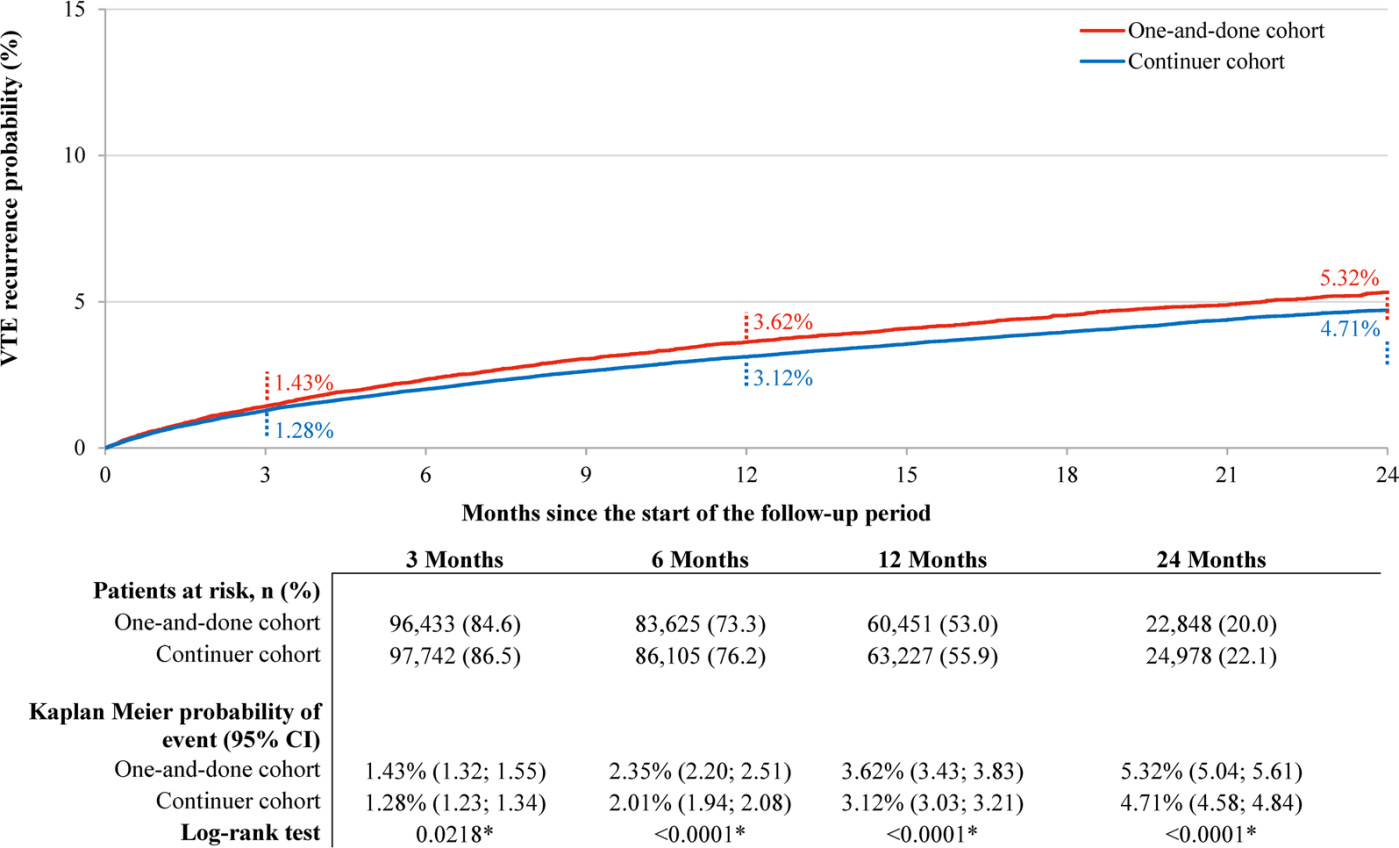
VTE recurrence (DVT or PE) probability in weighted one-and-done versus continuer cohorts (sensitivity analysis using a 60-day landmark period).^a^ *CI* Confidence interval; *DOAC* Direct oral anticoagulant; *DVT* deep-vein thrombosis; *PE* Pulmonary embolism; *VTE* Venous thromboembolism. *Note*: (a) VTE recurrence was identified in an inpatient setting. The time to the first recurrent event was measured from day 61 post-index date (i.e., first day after the end of the landmark period); patients for whom the event was not observed during the follow-up period were censored at the end of the follow-up period

**Fig. 5:**
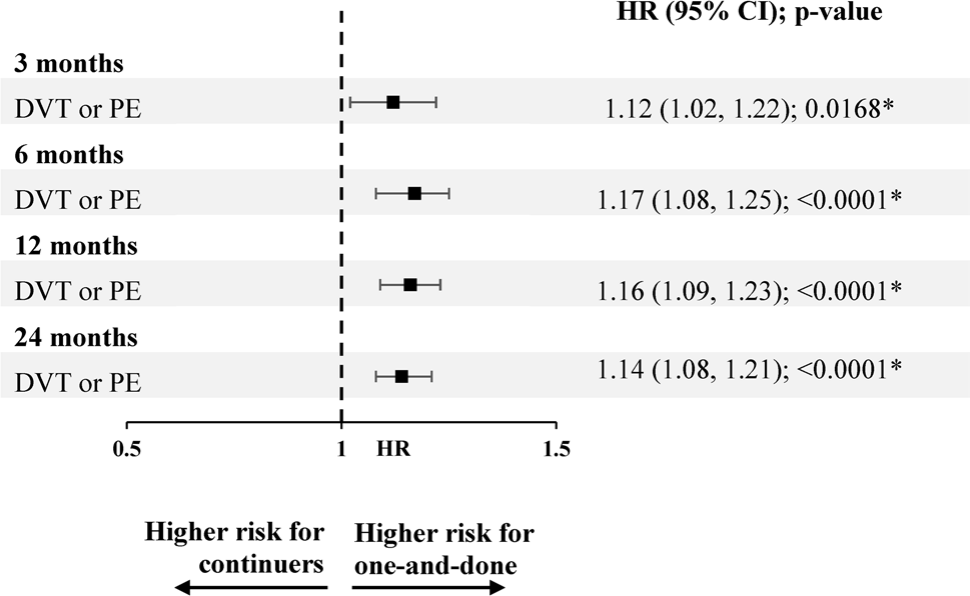
Risk of VTE recurrence in weighted one-and-done versus continuer cohorts (sensitivity analysis using a 60-day landmark period).^a^ *p-value < 0.05; *CI* Confidence interval; *DVT* Deep-vein thrombosis; *HR* Hazard ratio; *PE* Pulmonary embolism; *VTE* Venous thromboembolism. *Note*: (a) VTE recurrence was identified in an inpatient setting. HRs were generated using univariate weighted Cox proportional hazard models

A total of 114,031 patients were included in the weighted one-and-done cohort and 113,024 patients in the weighted continuer cohort using a 60-day landmark period. The probabilities of VTE recurrence in the two cohorts were comparable to those of the main analysis (Fig. 4). There was a 12–17% higher risk of VTE recurrence (composite outcome) in the one-and-done cohort compared to the continuer cohort at 3, 6, 12, and 24 months of follow-up (*p*-value at 3 months = 0.0168; all remaining *p*-values < 0.0001; Fig. 5).

## Discussion

This retrospective, real-world study demonstrated that 27.0% of patients discontinued DOAC treatment after filling their first prescription (“one-and-done”). These patients had a significantly higher risk of VTE recurrence (as a composite outcome and as DVT and PE separately) in both the short and long term, with a 16% higher risk at 3 months and 19% higher risk at 12 months of follow-up compared to patients who continued DOAC treatment beyond the first fill. The findings remained significant when extending the landmark period to 60 days. While the longer landmark period resulted in some earlier recurrences not being captured (i.e., during days 45–60), the higher risk of recurrence from longer time off DOAC treatment in the one-and-done cohort led to similar, albeit on the lower end, probabilities of recurrence compared to the main analysis. These slightly lower rates may be explained by the fact that the risk of VTE recurrence is typically highest within the first 6 months of the incident VTE, with a steep increase in recurrences between days 0–60 [19], and some recurrent events may not have been captured during the longer landmark period.

This study is subject to some limitations. Since the database does not capture services received from providers outside of the network, the use of treatments and healthcare services may be underestimated. For instance, if patients changed claims transaction networks for their prescriptions after the first fill, they may have appeared to discontinue DOAC therapy. A filled prescription did not guarantee that the medication was taken as prescribed; therefore, treatment continuation may have been overestimated. Furthermore, patients with only one approved DOAC claim may have received free drug samples, which would not have been captured in the data. However, this would have biased the results toward the null and led to a more conservative effect size. The requirement of ≥ 45 days post-index (i.e., landmark period) introduced a selection and survival bias; specifically, only patients without VTE recurrence during the landmark period were included in both cohorts, and the one-and-done cohort may have been less likely to survive beyond 45 days than the continuer cohort. Therefore, the landmark period may have selected for patients with less severe VTE and longer survival in the one-and-done cohort compared to the continuer cohort. While the database covers > 75% of US retail prescription claims, generalizability to the total US population was not evaluated in this study. Lastly, this study may have been subject to residual confounding due to unmeasured confounders.

To our knowledge, this is the first study evaluating the discontinuation of DOACs after the first prescription filled. The American Society of Hematology guidelines recommend a minimum of 3–6 months of anticoagulant therapy for new VTE [7]. Since the present study evaluated discontinuation after up to 30 days of treatment, not requesting a refill was a more likely reason for discontinuation than coming to an end of prescribed treatment. However, reasons for discontinuation were not available in the data and should be assessed in future studies.

Given that more than one-quarter of patients with VTE discontinued DOACs after the first fill, the current study addresses an important knowledge gap regarding the clinical consequences of this common treatment pattern. Prior literature evaluated clinical implications of DOAC nonadherence and non-persistence in VTE, and while the studied measures of nonadherence and non-persistence were different from the measure of DOAC discontinuation after up to 30 days of treatment in the current study, they were associated with similar clinical consequences. Studies that focused on patients with ≥ 3 months of initial VTE treatment demonstrated a link between DOAC nonadherence and VTE recurrence [4, 5, 20]. Furthermore, lack of DOAC persistence in the first 3 months has also been associated with increased risk of hospital or emergency room readmission [13]. Notably, Patel et al. showed that participation in a pharmacist-led DOAC education class was associated with improved persistence [13]. Together with these prior publications, the current study suggests that persistence on DOACs is essential both in the first 30 days of therapy and later to prevent VTE recurrence and other negative clinical consequences.

The clinical implications of VTE recurrence after DOAC discontinuation can be costly, both medically and economically. For instance, in a meta-analysis of randomized controlled trials and prospective cohort studies, the pooled fatality rate of recurrent VTE after discontinuation of anticoagulation was 3.8% [20]. In addition, recurrent PE and DVT have both been found to be significantly associated with subsequent risk of death in a separate study [21]. Regarding economic outcomes, patients with VTE recurrence have higher rates of hospitalizations and emergency department visits, as well as higher healthcare costs, compared to patients without VTE recurrence [22, 23]. Therefore, there is an urgent need to understand the factors associated with early DOAC discontinuation so that VTE management can be optimized, potentially reducing the rates of VTE recurrence and the subsequent clinical and economic implications.

## Conclusion

In this retrospective, real-world study, a substantial proportion of patients with VTE discontinued DOACs after up to one month of therapy (i.e., after the first DOAC claim; “one-and-done”), and these patients had significantly higher probability of a recurrent VTE event than patients who continued DOAC therapy.

## Supplementary Information

Below is the link to the electronic supplementary material.

Supplementary file1 (DOCX 122 KB)
