# Supplementary material for: Venous thromboembolism recurrence among one-and-done direct oral anticoagulant users: a retrospective longitudinal study
Source: Int J Clin Pharm. 2023 May 19;45(4):952–61. doi: 10.1007/s11096-023-01589-7 (PMC10366276; doi:10.1007/s11096-023-01589-7)
Supplement: Supplementary file 1 — Supplementary file1 (DOCX 122 KB) [file 11096_2023_1589_MOESM1_ESM.docx]

Supplementary materials

Article title: Venous thromboembolism recurrence among one-and-done direct oral anticoagulant users: a retrospective longitudinal study

*International Journal of Clinical Pharmacy*

**Authors:** Mark Alberts, MD; Maryia Zhdanava, MA; Dominic Pilon, MA; Gabrielle Caron-Lapointe, BSc; Patrick Lefebvre, MA; Brahim Bookhart, MBA, MPH; Akshay Kharat, PhD

**Corresponding author:**

Maryia Zhdanava, MA

Manager

Analysis Group, Inc.

1190 avenue des Canadiens-de-Montréal, Suite 1500

Montréal, QC H3B 0G7, Canada

Phone: 514-394-4469

E-mail: [Masha.Zhdanava@analysisgroup.com](mailto:Masha.Zhdanava@analysisgroup.com)

Supplementary Figure 1. Study design

**6-month baseline period**

Description of patient characteristics

Clinical activity or data start

Earliest date between end of data availability and end of clinical activity

**Follow-up period**

Evaluation of VTE recurrence

**Index date**
First DOAC claim (approved only)

**45-day landmark period**

(Sensitivity: 60 days)

DOAC = direct oral anticoagulant; VTE = venous thromboembolism.

Supplementary Table 1. Diagnosis codes used in the study

| **Medical condition** | **ICD-9-CM** | **ICD-10-CM** |
| --- | --- | --- |
| **Stroke and VTE** |  |  |
| Ischemic stroke | 433.x1, 434.x1, 436 | I63.xx |
| Hemorrhagic Stroke | 430.xx, 431.xx, 432.9x | I60.xx, I61.x, I62.xx |
| DVT | 451.1, 451.2, 453.4, 453.8, 453.9 | I80.1, I80.2, I80.3, 182.4, I82.6, I82.A1, I82.B1, I82.C1, I82.90 |
| PE | 415.1 | I26.x |
| **Exclusion criteria** |  |  |
| Atrial fibrillation | 427.31 | I48.x |
| Organ or tissue replaced by transplant | V42.x | Z48.2, Z94.x, Z95.3 |
| Pregnancy | V22.x | Z33.x, Z34.xx |
| **Baseline diagnoses and risk factors** |  |  |
| Solid cancers | 140.xx-149.xx, 150.xx-159.xx, 160.xx-165.xx, 170.xx-176.xx, 179.xx-189.xx, 190.xx-195.xx, 199.x | C00.xxx-C14.xxx, C15.xxx-C26.xxx, C30.xxx-C39.xxx, C40.xxx-C41.xxx, C43.xxx-C4A.xxx, C45.xxx-C49.xxx, C50.xxx, C51.xxx-C58.xxx, C60.xxx-C63.xxx, C64.xxx-C68.xxx, C69.xxx-C72.xxx, C73.xxx-C75.xxx, C76.xxx, C80.xxx |
| Metastasis cancer | 196.xx-198.x | C77.xxx-C79.xxx |
| Hematologic cancers | 200.xx-208.xx | C81.xxx-C96.xxx |
| Hypertension | 401.x-405.x | I10, I11.x, I12.x, I13.x, I15.x |
| Hyperlipidemia | 272.0, 272.1, 272.2, 272.3,272.4 | E78.0, E78.1, E78.2, E78.3, E78.4, E78.5 |
| Diabetes | 250.xx | E10.x, E11.x, E13.x |
| Other serious infections | 001.x-139.x | A00.x-A99, B00.x-B99.x |
| Obesity | 278.00, 278.01, 278.03, V85.3, V85.4 | E66.0, E66.1, E66.2, E66.8, E66.9 |
|  |  |  |

DVT = deep-vein thrombosis; ICD-9-CM/ICD-10-CM = International Classification of Disease, Ninth/Tenth Revision, Clinical Modification; PE = pulmonary embolism; VTE = venous thromboembolism.

Supplementary Table 2. Drug codes for anti-cancer treatments

| **Drug^1^** | **GPI** | **HCPCS** |
| --- | --- | --- |
| *Checkpoint Inhibitors* |  |  |
| Atezolizumab | 2135301500 | C9483, J9022 |
| Avelumab | 2135302000 | C9491, J9023 |
| Durvalumab | 2135302900 | C9492, J9173 |
| Ipilimumab | 2135303200 | C9284, J9228 |
| Nivolumab | 2135304100 | C9453, J9299 |
| Pembrolizumab | 2135305300 | C9027, J9271 |
| *Other Biologic Treatments* |  |  |
| Aflibercept | 2133501010 | Q2046, J0178, C9291, C9296, J9400 |
| Alemtuzumab | 2135301000 | Q9979, J9010, J0202 |
| Bevacizumab | 2133502000, 2133502020 | J9035, S0116, Q2024, Q5107, C9257, C9214 |
| Blinatumomab | 2135202000 | C9449, J9039 |
| Brentuximab | 2135502020 | J9042, C9287 |
| Cemiplimab | 2135302340 |  |
| Cetuximab | 2135302500 | C9215, J9055 |
| Daratumumab | 2135302700 | C9476, J9145 |
| Dinutuximab | 2135302800 |  |
| Elotuzumab | 2135303000 | C9477, J9176 |
| Gemtuzumab | 2135503020 | J9300, J9203 |
| Ibritumomab | 2135803520, 2135803540 | A9542, A9543 |
| Inotuzumab | 2135504020 | C9028, J9229 |
| Mogamulizumab | 2135303520 |  |
| Moxetumomab | 2135303650 |  |
| Necitumumab | 2135303800 | C9475, J9295 |
| Obinutuzumab | 2135304300 | C9021, J9301 |
| Ofatumumab | 2135304500 | C9260, J9302 |
| Olaratumab | 2135304700 | C9485, J9285 |
| Panitumumab | 2135305000 | C9235, J9303 |
| Pertuzumab | 2135305400 | C9292, J9306 |
| Polatuzumab Vedotin | 2135506030 |  |
| Ramucirumab | 2133507000 | C9025, J9308 |
| Rituximab | 2135306000, 2199000264, 2135306010 | J9310, J9312 |
| Tositumomab | 2135807000, 2160004000 | A9545, G3001, A9544 |
| Trastuzumab | 2135307000, 2135507030, 2135307014, 2135307030, 2199000272 | C9131, J9354, J9355, G9835 |
| *Small Molecule Chemotherapies* |  |  |
| Abemaciclib | 2153101000 |  |
| Acalabrutinib | 2153400300 |  |
| Afatinib | 2153400610 |  |
| Aldesleukin | 2170302000 | J9015 |
| Alectinib | 2153400710 |  |
| Alpelisib | 2153801000 |  |
| Altretamine | 2110000500 |  |
| Arsenic | 2170000810 | J9017 |
| Asparaginase | 2125001000, 2125001040 | J9020, J9019, C9289 |
| Axitinib | 2153400800 |  |
| Azacitidine | 2130000300 | J9025, C9218, S0168 |
| Belinostat | 2153152000 | C9442, J9032 |
| Bendamustine | 2110000910 | C9243, J9033, J9034 |
| Bexarotene | 2170822000 |  |
| Bicalutamide | 2140242000 |  |
| Binimetinib | 2153352000 |  |
| Bleomycin | 2120001010 | C9417, J9040 |
| Bortezomib | 2153601500 | J9041, J9044, S0115, C9207 |
| Bosutinib | 2153401200 |  |
| Brigatinib | 2153401000 |  |
| Busulfan | 2110001000 | C1178, J0594, J8510 |
| Cabazitaxel | 2150000300 | C9276, J9043 |
| Cabozantinib | 2153401310 |  |
| Capecitabine | 2130000500 | J8520, J8521 |
| Carboplatin | 2110001500 | J9045 |
| Carfilzomib | 2153602500 | C9295, J9047 |
| Carmustine | 2110201000, 2110201020 | J9050, C9437 |
| Ceritinib | 2153401400 |  |
| Chlorambucil | 2110101000 | S0172 |
| Cisplatin | 2110002000 | C9418, J9060, J9062 |
| Cladribine | 2130000700 | C9419, J9065 |
| Clofarabine | 2130000800 | J9027, C9129 |
| Cobimetinib | 2153353020 |  |
| Copanlisib | 2153802010 | C9030, J9057 |
| Crizotinib | 2153401500 |  |
| Cyclophosphamide | 2110102000 | C9420, C9421, J8530, J9070, J9080, J9090, J9091, J9092, J9093, J9094, J9095, J9096, J9097 |
| Cytarabine | 2130001000, 2130001030 | C9422, J9098, J9100, J9110 |
| Cytarabine/Daunorubicin | 2199000220 | C9024, J9153 |
| Dabrafenib | 2153202510 |  |
| Dacarbazine | 2170002000 | C9423, J9130, J9140 |
| Dacomitinib | 2153401900 |  |
| Dactinomycin | 2120002000 | J9120 |
| Dasatinib | 2153402000 |  |
| Daunorubicin | 2120003005, 2120003010 | C9424, J9150, J9151 |
| Decitabine | 2130001500 | J0894, C9231 |
| Denileukin | 2170002400 | J9160 |
| Docetaxel | 2150000500 | J9170, J9171 |
| Doxorubicin | 2120004010, 2120004040 | J9000, J9001, Q2050, Q2048, J9002, Q2049, C9415 |
| Duvelisib | 2153803000 |  |
| Enasidenib | 2153503020 |  |
| Encorafenib | 2153204000 |  |
| Entrectinib | 2153382000 |  |
| Enzalutamide | 2140243000 |  |
| Epirubicin | 2120004210 | J9178 |
| Erdafitinib | 2153222500 |  |
| Eribulin | 2150000920 | J9179, C9280 |
| Erlotinib | 2153402500, 2153402510 |  |
| Etoposide | 2150001000, 2150001060 | C9414, C9425, J8560, J9181, J9182 |
| Everolimus | 2153253000 | J8561, J7527 |
| Fedratinib | 2153752020 |  |
| Floxuridine | 2130002000 | C9426, J9200 |
| Fludarabine | 2130002510 | J9185, C9262, Q2025, J8562 |
| Fluorouracil | 2130003000 | J9190, S3722 |
| Flutamide | 2140244000 | S0175 |
| Fulvestrant | 2140353000 | J9395 |
| Gefitinib | 2153403000 | J8565 |
| Gemcitabine | 2130003410, 2130003411 | J9201 |
| Gilteritinib | 2153403120 |  |
| Glasdegib | 2137003030 |  |
| Goserelin | 2140500510 | J9202 |
| Hydroxyurea | 2170003000 | S0176 |
| Ixazomib | 2153604510 |  |
| Ibrutinib | 2153403300 |  |
| Idarubicin | 2120004510 | C9429, J9211 |
| Idelalisib | 2153804000 |  |
| Ifosfamide | 2110102500 | C9427, J9208 |
| Ifosfamide/mesna | 2199000240 |  |
| Imatinib | 2153403510 | S0088 |
| Interferon alfa-2a | 2170006010 | J9213 |
| Interferon alfa-2b | 2170006020 | J9214 |
| Interferon alpha-n3 | 2170006030 | J9215 |
| Interferon gamma-1b | 2170006070 | J9216 |
| Irinotecan | 2155004010, 2155004020 | J9206, C9474, J9205 |
| Ivosidenib | 2153494000 |  |
| Ixabepilone | 2150001100 | J9207, C9240 |
| Lapatinib | 2153405010 |  |
| Larotrectinib | 2153383520 |  |
| Lenvatinib | 2153405420 |  |
| Lomustine | 2110202000 | S0178 |
| Lorlatinib | 2153405600 |  |
| Mechlorethamine | 2110103010 | J9230 |
| Melphalan | 2110104000, 2110104010 | J8600, J9245 |
| Mercaptopurine | 2130004000 | S0108 |
| Mesna | 2175805000 | C9428, J9209 |
| Methotrexate | 2130005000, 2130005010 | J8610, J9250, J9260 |
| Midostaurin | 2153303000 |  |
| Mitomycin | 2120005000 | C9432, J9280, J9290, J7315 |
| Mitoxantrone | 2120005500 | J9293 |
| Nelarabine | 2130005200 | J9261 |
| Neratinib | 2153405810 |  |
| Nilotinib | 2153406000, 2153406020 |  |
| Niraparib | 2153555020 |  |
| Olaparib | 2153556000 |  |
| Omacetaxine | 2170004010 | C9297, J9262 |
| Osimertinib | 2153406520 |  |
| Oxaliplatin | 2110002800 | C9205, J9263 |
| Paclitaxel | 2150001200, 2150001220 | C9127, C9431, J9264, J9265, J9267 |
| Palbociclib | 2153106000 |  |
| Pazopanib | 2153407010 |  |
| Pegaspargase | 2125006000 | J9266 |
| Peginterferon alfa-2b | 2170007520 | S0146, S0148 |
| Pemetrexed | 2130005310 | J9305, C9213 |
| Pentostatin | 2170004500 | J9268 |
| Pexidartinib | 2153407301 |  |
| Pipobroman | 2110003000 |  |
| Plicamycin | 2120006000 | J9270 |
| Pomalidomide | 2145008000 |  |
| Ponatinib | 2153407510 |  |
| Porfimer | 2170707010 | J9600 |
| Pralatrexate | 2130005400 | C9259, J9307 |
| Procarbazine | 2170005010 | S0182 |
| Regorafenib | 2153305000 |  |
| Romidepsin | 2153156000 | C9265, J9315 |
| Rucaparib | 2153557020 |  |
| Ruxolitinib | 2153756020 |  |
| Selinexor | 2156006000 |  |
| Sonidegib | 2137006020 |  |
| Sorafenib | 2153306040 |  |
| Streptozocin | 2110203000 | J9320 |
| Sunitinib | 2153307030 |  |
| Talazoparib | 2153558040 |  |
| Temozolomide | 2110407000 | J8700, J9328, C9253 |
| Temsirolimus | 2153257000 | J9330, C9239 |
| Teniposide | 2150001500 | Q2017 |
| Thioguanine | 2130006000 |  |
| Thiotepa | 2110004000 | C9433, J9340 |
| Trifluridine/tipiracil | 2199000275 |  |
| Topotecan | 2155008010 | J9350, J8705, J9351 |
| Trabectedin | 2110707500 | C9480, J9352 |
| Trametinib | 2153357010 |  |
| Uracil | 2110105000 |  |
| Valrubicin | 2120008000 | J9357 |
| Vandetanib | 2153408500 |  |
| Vemurafenib | 2153208000 |  |
| Venetoclax | 2147008000 |  |
| Vinblastine | 2150003010 | J9360 |
| Vincristine | 2150002010, 2150002020 | J9370, J9375, J9380, J9371 |
| Vinorelbine | 2150005080 | C9440, J9390 |
| Vismodegib | 2137007000 |  |
| Vorinostat | 2153157500 |  |
| Zanubrutinib | 2153409500 |  |
| *Other Immunologic Therapies* |  |  |
| Axicabtagene Ciloleucel | 2165101010 | Q2041 |
| BCG live | 2170001300 | J9031 |
| Sipuleucel | 2165107000 | C9273, Q2043 |
| Talimogene | 2157407040 | C9472, J9325 |
| Tisagenlecleucel | 2165107500 | Q2040 |
| *Hormone Therapies* |  |  |
| Abarelix | 2140551500 | C9216, J0128, S0165 |
| Abiraterone | 2140601020 |  |
| Anastrozole | 2140281000 | S0170 |
| Apalutamide | 2140241000 |  |
| Darolutamide | 2140242500 |  |
| Degarelix | 2140552510 | J9155 |
| Diethylstilbestrol | 2140301010 | C9439, J9165 |
| Exemestane | 2140283500 | S0156 |
| Histrelin | 2140500710 | J1675, Q2020, S0133, J9225, J9226 |
| Hydroxyprogesterone | 2140400720 | J1725, J1726, J1729, Q9985, Q9986 |
| Letrozole | 2140286000 |  |
| Leuprolide | 2140501010, 2140501015, 2140501020, 2140501025, 2140990260 | C9430, J1950, J9217, J9218, J9219 |
| Medroxyprogesterone | 2140401010 | J1051, J1050, J1056, J1055 |
| Megestrol | 2140402010 | S0179 |
| Nilutamide | 2140246000 |  |
| Polyestradiol | 2140303010 |  |
| Tamoxifen | 2140268010 | S0187, G8376 |
| Testolactone | 2140202000 |  |
| Toremifene | 2140268510 |  |
| Tretinoin | 2170808000 | S0117 |
| Triptorelin | 2140505020 | J3315, C9016 |
| *Radioactive Therapies* |  |  |
| Iobenguane | 2160003520 | A9508, A9582 |
| Lutetium Dotatate | 2160004520 | A9513, C9031 |
| Radium 223 | 2160005500 | A9606 |
| Samarium | 2160006500 | A9605, A9604 |
| Strontium 89 | 2160007000 | A9600, C9401 |
| *Other Therapies* |  |  |
| Amifostine | 2175801000, 2175801010 | J0207 |
| Calaspargase Pegol | 2125003050 |  |
| Dexrazoxane | 2175404000, 2175404010 | J1190 |
| Estramustine | 2140302010 |  |
| Glucarpidase | 2175603000 | C9293 |
| Leucovorin | 2175504010, 2175504060 | J0640 |
| Levamisole | 2145006010 | S0177 |
| Levoleucovorin | 2175505010, 2175505040, 2175505000 | J0641 |
| Methoxsalen | 2170705000 |  |
| Mitotane | 2140225000 |  |
| Palifermin | 2176506000 | J2425 |
| Panobinostat | 2153155010 |  |
| Rasburicase | 2176406500 | J2783 |
| Ribociclib | 2153107050 |  |
| Ribociclib/Succinate-Letrozole | 2199000260 |  |
| Tagraxofusp-erzs | 2170308030 |  |

GPI = generic product identifier; HCPCS = healthcare common procedure coding system.

Supplementary Table 3. Procedure codes for radiation cancer therapy

| **Code** | **Description** | **Code Type** |
| --- | --- | --- |
| 0182T | High dose rate electronic brachytherapy, per fraction | CPT |
| 0190T | Placement of intraocular radiation source applicator (List separately in addition to primary procedure) | CPT |
| 0197T | Intra-fraction localization and tracking of target or patient motion during delivery of radiation therapy (eg, 3D positional tracking, gating, 3D surface tracking), each fraction of treatment | CPT |
| 19296 | Insertion of single channel expandable afterloading brachytherapy catheter for radiotherapy into breast using imaging guidance | CPT |
| 19296 | Insertion of multi channel expandable afterloading brachytherapy catheter for radiotherapy into breast using imaging guidance | CPT |
| 19297 | Insertion of multi channel expandable afterloading brachytherapy catheter for radiotherapy into breast using imaging guidance | CPT |
| 19297 | Insertion of single channel expandable afterloading brachytherapy catheter for radiotherapy into breast using imaging guidance | CPT |
| 19298 | Insertion of afterloading button type and multitube brachytherapy catheter for radiotherapy into breast using imaging guidance | CPT |
| 20555 | Insertion of catheter into muscle for radiation therapy | CPT |
| 20555 | Insertion of needle into soft tissue for radiation therapy | CPT |
| 20555 | Insertion of catheter into muscle and soft tissue for radiation therapy | CPT |
| 20555 | Insertion of needle into muscle for radiation therapy | CPT |
| 20555 | Insertion of needle into muscle and soft tissue for radiation therapy | CPT |
| 20555 | Insertion of catheter into soft tissue for radiation therapy | CPT |
| 20982 | Percutaneous radiofrequency ablation of tumor of bone using computed tomography guidance | CPT |
| 31643 | Flexible bronchoscopy with placement of catheter for intracavitary radioelement application | CPT |
| 31643 | Flexible bronchoscopy with placement of catheter for intracavitary radioelement application using fluoroscopic guidance | CPT |
| 31643 | Rigid bronchoscopy with placement of catheter for intracavitary radioelement application | CPT |
| 31643 | Rigid bronchoscopy with placement of catheter for intracavitary radioelement application using fluoroscopic guidance | CPT |
| 32553 | Percutaneous placement of intrathoracic interstitial device for radiation therapy guidance | CPT |
| 32701 | Thoracic target delineation for stereotactic body radiation therapy | CPT |
| 32998 | Unilateral percutaneous radiofrequency ablation therapy for reduction of pulmonary tumor including chest wall | CPT |
| 32998 | Unilateral percutaneous radiofrequency ablation therapy for reduction of pulmonary tumor | CPT |
| 32998 | Unilateral percutaneous radiofrequency ablation therapy for eradication of pulmonary tumor including pleura | CPT |
| 32998 | Unilateral percutaneous radiofrequency ablation therapy for eradication of pulmonary tumor including chest wall | CPT |
| 32998 | Unilateral percutaneous radiofrequency ablation therapy for reduction of pulmonary tumor including pleura | CPT |
| 32998 | Unilateral percutaneous radiofrequency ablation therapy for eradication of pulmonary tumor | CPT |
| 41019 | Transnasal placement of catheter into head and neck region for interstitial radioelement application | CPT |
| 41019 | Percutaneous placement of catheter into head and neck region for interstitial radioelement application | CPT |
| 41019 | Percutaneous placement of needle into head for interstitial radioelement application | CPT |
| 41019 | Transnasal placement of catheter into neck for interstitial radioelement application | CPT |
| 41019 | Percutaneous placement of catheter into head for interstitial radioelement application | CPT |
| 41019 | Percutaneous placement of needle into neck for interstitial radioelement application | CPT |
| 41019 | Percutaneous placement of needle into head and neck region for interstitial radioelement application | CPT |
| 41019 | Percutaneous placement of catheter into neck for interstitial radioelement application | CPT |
| 41019 | Transoral placement of needle into neck for interstitial radioelement application | CPT |
| 41019 | Transoral placement of catheter into head for interstitial radioelement application | CPT |
| 41019 | Transoral placement of catheter into head and neck region for interstitial radioelement application | CPT |
| 41019 | Transoral placement of needle into head and neck region for interstitial radioelement application | CPT |
| 41019 | Transoral placement of catheter into neck for interstitial radioelement application | CPT |
| 41019 | Transnasal placement of needle into head and neck region for interstitial radioelement application | CPT |
| 41019 | Transoral placement of needle into head for interstitial radioelement application | CPT |
| 41019 | Transnasal placement of needle into neck for interstitial radioelement application | CPT |
| 41019 | Transnasal placement of catheter into head for interstitial radioelement application | CPT |
| 41019 | Transnasal placement of needle into head for interstitial radioelement application | CPT |
| 4165F | 3-dimensional conformal radiotherapy (3D-CRT) or intensity modulated radiation therapy (IMRT) received (PRCA) | CPT |
| 4181F | Conformal radiation therapy received (NMA-No Measure Assoc.) | CPT |
| 4200F | External beam radiotherapy as primary therapy to prostate with or without nodal irradiation (PRCA) | CPT |
| 4201F | External beam radiotherapy with or without nodal irradiation as adjuvant or salvage therapy for prostate cancer patient (PRCA) | CPT |
| 47370 | Surgical laparoscopy with radiofrequency ablation of liver tumor | CPT |
| 47380 | Open radiofrequency ablation of liver tumor | CPT |
| 47382 | Percutaneous radiofrequency ablation of liver tumor | CPT |
| 49327 | Surgical laparoscopy with retroperitoneal placement of interstitial device for radiation therapy guidance using imaging guidance | CPT |
| 49327 | Surgical laparoscopy with retroperitoneal placement of interstitial device for radiation therapy guidance | CPT |
| 49327 | Surgical laparoscopy with intra-pelvic placement of interstitial device for radiation therapy guidance using imaging guidance | CPT |
| 49327 | Surgical laparoscopy with intra-pelvic placement of interstitial device for radiation therapy guidance | CPT |
| 49327 | Surgical laparoscopy with intra-pelvic and retroperitoneal placement of interstitial device for radiation therapy guidance using imaging guidance | CPT |
| 49327 | Surgical laparoscopy with intra-pelvic and retroperitoneal placement of interstitial device for radiation therapy guidance | CPT |
| 49327 | Surgical laparoscopy with intra-abdominal placement of interstitial device for radiation therapy guidance using imaging guidance | CPT |
| 49327 | Surgical laparoscopy with intra-abdominal placement of interstitial device for radiation therapy guidance | CPT |
| 49327 | Surgical laparoscopy with intra-abdominal and retroperitoneal placement of interstitial device for radiation therapy guidance using imaging guidance | CPT |
| 49327 | Surgical laparoscopy with intra-abdominal and retroperitoneal placement of interstitial device for radiation therapy guidance | CPT |
| 49327 | Surgical laparoscopy with intra-abdominal and intra-pelvic placement of interstitial device for radiation therapy guidance using imaging guidance | CPT |
| 49327 | Surgical laparoscopy with intra-abdominal and intra-pelvic placement of interstitial device for radiation therapy guidance | CPT |
| 49411 | Retroperitoneal placement of interstitial device for radiation therapy guidance | CPT |
| 49411 | Percutaneous placement of interstitial device for radiation therapy guidance | CPT |
| 49411 | Intra-pelvic placement of interstitial device for radiation therapy guidance | CPT |
| 49411 | Intra-abdominal placement of interstitial device for radiation therapy guidance | CPT |
| 49412 | Open intra-abdominal and intrapelvic placement of interstitial device for radiation therapy guidance using imaging guidance | CPT |
| 49412 | Open intra-abdominal and intrapelvic placement of interstitial device for radiation therapy guidance | CPT |
| 49412 | Open retroperitoneal placement of interstitial device for radiation therapy guidance using imaging guidance | CPT |
| 49412 | Open retroperitoneal placement of interstitial device for radiation therapy guidance | CPT |
| 49412 | Open intrapelvic placement of interstitial device for radiation therapy guidance using imaging guidance | CPT |
| 49412 | Open intrapelvic placement of interstitial device for radiation therapy guidance | CPT |
| 49412 | Open intrapelvic and retroperitoneal placement of interstitial device for radiation therapy guidance using imaging guidance | CPT |
| 49412 | Open intrapelvic and retroperitoneal placement of interstitial device for radiation therapy guidance | CPT |
| 49412 | Open intra-abdominal, intrapelvic and retroperitoneal placement of interstitial device for radiation therapy guidance using imaging guidance | CPT |
| 49412 | Open intra-abdominal, intrapelvic and retroperitoneal placement of interstitial device for radiation therapy guidance | CPT |
| 49412 | Open intra-abdominal placement of interstitial device for radiation therapy guidance using imaging guidance | CPT |
| 49412 | Open intra-abdominal placement of interstitial device for radiation therapy guidance | CPT |
| 50592 | Unilateral percutaneous radiofrequency ablation of renal tumor | CPT |
| 53852 | Destruction of prostate tissue using radiofrequency thermotherapy by transurethral approach | CPT |
| 55875 | Transperineal placement of needle into prostate for application of interstitial radioelement, with cystoscopy | CPT |
| 55875 | Transperineal placement of needle into prostate for application of interstitial radioelement | CPT |
| 55876 | Placement of interstitial device in prostate for radiation therapy guidance | CPT |
| 55920 | Placement of needle into pelvic organ for application of interstitial radioelement | CPT |
| 55920 | Placement of needle into genital for application of interstitial radioelement | CPT |
| 55920 | Placement of needle into genital for application of interstitial radioelement | CPT |
| 55920 | Placement of catheter into genital for application of interstitial radioelement | CPT |
| 57155 | Insertion of vaginal ovoid for clinical brachytherapy | CPT |
| 57155 | Insertion of uterine tandem for clinical brachytherapy | CPT |
| 57155 | Insertion of uterine tandem and vaginal ovoid for clinical brachytherapy | CPT |
| 57156 | Insertion of vaginal radiation afterloading apparatus for clinical brachytherapy | CPT |
| 58346 | Insertion of Heyman capsule for clinical brachytherapy | CPT |
| 61720 | Creation of lesion of thalamus by stereotactic method with burr hole and localizing and recording techniques | CPT |
| 61720 | Creation of lesion of thalamus by stereotactic method | CPT |
| 61720 | Creation of lesion of globus pallidus by stereotactic method with burr hole and localizing and recording techniques | CPT |
| 61720 | Creation of lesion of globus pallidus by stereotactic method | CPT |
| 61735 | Creation of lesion of subcortical structure by stereotactic method with burr hole and localizing and recording techniques | CPT |
| 61735 | Creation of lesion of subcortical structure by stereotactic method | CPT |
| 61770 | Localization by stereotactic method with insertion of probe for placement of radiation source | CPT |
| 61770 | Localization by stereotactic method with insertion of catheter for placement of radiation source | CPT |
| 61770 | Localization by stereotactic method with burr hole and insertion of probe for placement of radiation source | CPT |
| 61770 | Localization by stereotactic method with burr hole and insertion of catheter for placement of radiation source | CPT |
| 61781 | Stereotactic computer-assisted cranial intradural procedure | CPT |
| 61782 | Stereotactic computer-assisted cranial extradural procedure | CPT |
| 61783 | Stereotactic computer-assisted spinal procedure | CPT |
| 61790 | Percutaneous creation of lesion of gasserian ganglion using neurolytic agent by stereotactic method | CPT |
| 61791 | Percutaneous creation of lesion of trigeminal medullary tract using neurolytic agent by stereotactic method | CPT |
| 61796 | Radiosurgery on cranial lesion by stereotactic method | CPT |
| 61797 | Radiosurgery on cranial lesion by stereotactic method | CPT |
| 61798 | Radiosurgery on cranial lesion by stereotactic method | CPT |
| 61799 | Radiosurgery on cranial lesion by stereotactic method | CPT |
| 61800 | Application of stereotactic headframe for stereotactic radiosurgery | CPT |
| 63620 | Radiosurgery of spinal lesion using particle beam by stereotactic method | CPT |
| 63620 | Radiosurgery of spinal lesion using linear accelerator by stereotactic method | CPT |
| 63620 | Radiosurgery of spinal lesion using gamma ray by stereotactic method | CPT |
| 63621 | Radiosurgery of spinal lesion using particle beam by stereotactic method | CPT |
| 63621 | Radiosurgery of spinal lesion using linear accelerator by stereotactic method | CPT |
| 63621 | Radiosurgery of spinal lesion using gamma ray by stereotactic method | CPT |
| 67218 | Radiation of lesion of retina by implantation of source | CPT |
| 76950 | Ultrasonic guidance for placement of radiation therapy fields | CPT |
| 76965 | Ultrasonic guidance for placement of radiation therapy fields | CPT |
| 77014 | CT guidance for placement of radiation therapy fields | CPT |
| 77300 | Basic radiation dosimetry calculation | CPT |
| 77301 | Intensity modulated radiotherapy plan | CPT |
| 77326 | Brachytherapy isodose plan | CPT |
| 77327 | Brachytherapy isodose plan | CPT |
| 77328 | Brachytherapy isodose plan | CPT |
| 77338 | Multi-leaf collimator devices, design and construction per IMRT plan | CPT |
| 77370 | Special medical radiation physics consultation | CPT |
| 77371 | Stereotactic radiosurgery radiation treatment delivery of cranial lesions | CPT |
| 77372 | Stereotactic radiosurgery radiation treatment delivery of cranial lesions | CPT |
| 77373 | Stereotactic radiosurgery radiation treatment delivery with image guidance | CPT |
| 77385 | Intensity modulated radiation treatment delivery (IMRT), includes guidance and tracking, when performed; simple | CPT |
| 77386 | Intensity modulated radiation treatment delivery (IMRT), includes guidance and tracking, when performed; complex | CPT |
| 77399 | Medical radiation special services | CPT |
| 77399 | Medical radiation physics services | CPT |
| 77399 | Medical radiation dosimetry and treatment devices | CPT |
| 77401 | Radiation treatment delivery, superficial and ortho voltage | CPT |
| 77401 | Radiation treatment delivery, superficial | CPT |
| 77401 | Radiation treatment delivery, ortho voltage | CPT |
| 77402 | Radiation treatment delivery of single treatment area | CPT |
| 77403 | Radiation treatment delivery of single treatment area | CPT |
| 77404 | Radiation treatment delivery of single treatment area | CPT |
| 77406 | Radiation treatment delivery of single treatment area | CPT |
| 77407 | Radiation treatment delivery of 2 separate treatment areas | CPT |
| 77408 | Radiation treatment delivery of 2 separate treatment areas | CPT |
| 77409 | Radiation treatment delivery of 2 separate treatment areas | CPT |
| 77411 | Radiation treatment delivery of 2 separate treatment areas | CPT |
| 77412 | Radiation treatment delivery of 3 or more separate treatment areas | CPT |
| 77413 | Radiation treatment delivery of 3 or more separate treatment areas | CPT |
| 77414 | Radiation treatment delivery of 3 or more separate treatment areas | CPT |
| 77416 | Radiation treatment delivery of 3 or more separate treatment areas | CPT |
| 77421 | Stereoscopic x-ray guidance for the delivery of radiation therapy | CPT |
| 77422 | High energy neutron radiation treatment delivery | CPT |
| 77423 | High energy neutron radiation treatment delivery | CPT |
| 77424 | X-ray of intraoperative radiation treatment delivery | CPT |
| 77425 | Intraoperative radiation treatment delivery of electrons | CPT |
| 77427 | Radiation treatment management | CPT |
| 77431 | Radiation treatment management | CPT |
| 77432 | Stereotactic radiation treatment management of cranial lesions | CPT |
| 77432 | Stereotactic radiation treatment management of cranial lesion | CPT |
| 77435 | Stereotactic body radiation therapy treatment management | CPT |
| 77469 | Intraoperative radiation treatment management | CPT |
| 77470 | Special treatment procedure (eg, total body irradiation, hemibody radiation, per oral or endocavitary irradiation) | CPT |
| 77750 | Infusion or instillation of radioelement solution | CPT |
| 77761 | Intracavitary radiation source application | CPT |
| 77762 | Intracavitary radiation source application | CPT |
| 77763 | Intracavitary radiation source application | CPT |
| 77776 | Interstitial radiation source application | CPT |
| 77777 | Interstitial radiation source application | CPT |
| 77778 | Interstitial radiation source application | CPT |
| 77785 | Remote afterloading high dose rate radionuclide brachytherapy | CPT |
| 77786 | Remote afterloading high dose rate radionuclide brachytherapy | CPT |
| 77787 | Remote afterloading high dose rate radionuclide brachytherapy | CPT |
| 77789 | Surface application of radiation source | CPT |
| 77790 | Supervision, handling and loading of radiation source | CPT |
| 77799 | Clinical brachytherapy procedure | CPT |
| 92974 | Transcatheter placement of radiation delivery device for subsequent coronary intravascular brachytherapy | CPT |
| A4650 | Implantable radiation dosimeter, each | HCPCS |
| A9517 | Iodine i-131 sodium iodide capsule(s), therapeutic, per millicurie | HCPCS |
| A9527 | Iodine i-125, sodium iodide solution, therapeutic, per millicurie | HCPCS |
| A9530 | Iodine i-131 sodium iodide solution, therapeutic, per millicurie | HCPCS |
| C1715 | Brachytherapy needle | HCPCS |
| C1716 | Brachytherapy source, non-stranded, gold-198, per source | HCPCS |
| C1717 | Brachytherapy source, non-stranded, high dose rate iridium-192, per source | HCPCS |
| C1719 | Brachytherapy source, non-stranded, non-high dose rate iridium-192, per source | HCPCS |
| C1720 | Brachytherapy source, palladium 103, per source | HCPCS |
| C1728 | Catheter, brachytherapy seed administration | HCPCS |
| C2616 | Brachytherapy source, non-stranded, yttrium-90, per source | HCPCS |
| C2633 | Brachytherapy source, cesium-131, per source | HCPCS |
| C2634 | Brachytherapy source, non-stranded, high activity, iodine-125, greater than 1.01 mci (nist), per source | HCPCS |
| C2635 | Brachytherapy source, non-stranded, high activity, palladium-103, greater than 2.2 mci (nist), per source | HCPCS |
| C2636 | Brachytherapy linear source, non-stranded, palladium-103, per 1 mm | HCPCS |
| C2637 | Brachytherapy source, non-stranded, ytterbium-169, per source | HCPCS |
| C2638 | Brachytherapy source, stranded, iodine-125, per source | HCPCS |
| C2639 | Brachytherapy source, non-stranded, iodine-125, per source | HCPCS |
| C2640 | Brachytherapy source, stranded, palladium-103, per source | HCPCS |
| C2641 | Brachytherapy source, non-stranded, palladium-103, per source | HCPCS |
| C2642 | Brachytherapy source, stranded, cesium-131, per source | HCPCS |
| C2643 | Brachytherapy source, non-stranded, cesium-131, per source | HCPCS |
| C2644 | Brachytherapy source, cesium-131 chloride solution, per millicurie | HCPCS |
| C2645 | Brachytherapy planar source, palladium-103, per square millimeter | HCPCS |
| C2698 | Brachytherapy source, stranded, not otherwise specified, per source | HCPCS |
| C2699 | Brachytherapy source, non-stranded, not otherwise specified, per source | HCPCS |
| C9725 | Placement of endorectal intracavitary applicator for high intensity brachytherapy | HCPCS |
| C9726 | Placement and removal (if performed) of applicator into breast for intraoperative radiation therapy, add-on to primary breast procedure | HCPCS |
| C9728 | Placement of interstitial device(s) for radiation therapy/surgery guidance (e.g., fiducial markers, dosimeter), for other than the following sites (any approach): abdomen, pelvis, prostate, retroperitoneum, thorax, single or multiple | HCPCS |
| G0173 | Linear accelerator based stereotactic radiosurgery, complete course of therapy in one session | HCPCS |
| G0251 | Linear accelerator based stereotactic radiosurgery, delivery including collimator changes and custom plugging, fractionated treatment, all lesions, per session, maximum five sessions per course of treatment | HCPCS |
| G0338 | Performed for highly conformal distributions, plan positional accuracy and … | HCPCS |
| G0339 | Image-guided robotic linear accelerator-based stereotactic radiosurgery, complete course of therapy in one session or first session of fractionated treatment | HCPCS |
| G0340 | Image-guided robotic linear accelerator-based stereotactic radiosurgery, delivery including collimator changes and custom plugging, fractionated treatment, all lesions, per session, second through fifth sessions, maximum five sessions per course of treatm | HCPCS |
| G0458 | Low dose rate (ldr) prostate brachytherapy services, composite rate | HCPCS |
| G6001 | Ultrasonic guidance for placement of radiation therapy fields | HCPCS |
| G6002 | Stereoscopic x-ray guidance for localization of target volume for the delivery of radiation therapy | HCPCS |
| G6003 | Radiation treatment delivery, single treatment area,single port or parallel opposed ports, simple blocks or no blocks: up to 5 mev | HCPCS |
| G6004 | Radiation treatment delivery, single treatment area,single port or parallel opposed ports, simple blocks or no blocks: 6-10 mev | HCPCS |
| G6005 | Radiation treatment delivery, single treatment area,single port or parallel opposed ports, simple blocks or no blocks: 11-19 mev | HCPCS |
| G6006 | Radiation treatment delivery, single treatment area,single port or parallel opposed ports, simple blocks or no blocks: 20 mev or greater | HCPCS |
| G6007 | Radiation treatment delivery, 2 separate treatment areas, 3 or more ports on a single treatment area, use of multiple blocks: up to 5 mev | HCPCS |
| G6008 | Radiation treatment delivery, 2 separate treatment areas, 3 or more ports on a single treatment area, use of multiple blocks: 6-10 mev | HCPCS |
| G6009 | Radiation treatment delivery, 2 separate treatment areas, 3 or more ports on a single treatment area, use of multiple blocks: 11-19 mev | HCPCS |
| G6010 | Radiation treatment delivery, 2 separate treatment areas, 3 or more ports on a single treatment area, use of multiple blocks: 20 mev or greater | HCPCS |
| G6011 | Radiation treatment delivery,3 or more separate treatment areas, custom blocking, tangential ports, wedges, rotational beam, compensators, electron beam; up to 5 mev | HCPCS |
| G6012 | Radiation treatment delivery,3 or more separate treatment areas, custom blocking, tangential ports, wedges, rotational beam, compensators, electron beam; 6-10 mev | HCPCS |
| G6013 | Radiation treatment delivery,3 or more separate treatment areas, custom blocking, tangential ports, wedges, rotational beam, compensators, electron beam; 11-19 mev | HCPCS |
| G6014 | Radiation treatment delivery,3 or more separate treatment areas, custom blocking, tangential ports, wedges, rotational beam, compensators, electron beam; 20 mev or greater | HCPCS |
| G6017 | Intra-fraction localization and tracking of target or patient motion during delivery of radiation therapy (eg,3d positional tracking, gating, 3d surface tracking), each fraction of treatment | HCPCS |
| Q3001 | Radioelements for brachytherapy, any type, each | HCPCS |
| S8049 | Intraoperative radiation therapy (single administration) | HCPCS |
| 92.2 | Infusion of liquid brachytherapy radioisotope | ICD-9-PCS |
| 92.21 | Superficial radiation | ICD-9-PCS |
| 92.22 | Orthovoltage radiation | ICD-9-PCS |
| 92.23 | Radioisotopic teleradiotherapy | ICD-9-PCS |
| 92.24 | Teleradiotherapy using photons | ICD-9-PCS |
| 92.25 | Teleradiotherapy using electrons | ICD-9-PCS |
| 92.26 | Teleradiotherapy of other particulate radiation | ICD-9-PCS |
| 92.27 | Implantation or insertion of radioactive elements | ICD-9-PCS |
| 92.28 | Injection or instillation of radioisotopes | ICD-9-PCS |
| 92.29 | Other radiotherapeutic procedure | ICD-9-PCS |
| 92.3 | Stereotactic radiosurgery, not otherwise specified | ICD-9-PCS |
| 92.31 | Single source photon radiosurgery | ICD-9-PCS |
| 92.32 | Multi-source photon radiosurgery | ICD-9-PCS |
| 92.33 | Particulate radiosurgery | ICD-9-PCS |
| 92.39 | Stereotactic radiosurgery, not elsewhere classified | ICD-9-PCS |
| 92.41 | Intra-operative electron radiation therapy | ICD-9-PCS |
| 08H031Z | Insertion of Radioactive Element into Right Eye, Percutaneous Approach | ICD-10-PCS |
| 08H0X1Z | Insertion of Radioactive Element into Right Eye, External Approach | ICD-10-PCS |
| 08H131Z | Insertion of Radioactive Element into Left Eye, Percutaneous Approach | ICD-10-PCS |
| 08H1X1Z | Insertion of Radioactive Element into Left Eye, External Approach | ICD-10-PCS |
| 0BH001Z | Insertion of Radioactive Element into Tracheobronchial Tree, Open Approach | ICD-10-PCS |
| 0BH031Z | Insertion of Radioactive Element into Tracheobronchial Tree, Percutaneous Approach | ICD-10-PCS |
| 0BH041Z | Insertion of Radioactive Element into Tracheobronchial Tree, Percutaneous Endoscopic Approach | ICD-10-PCS |
| 0BH071Z | Insertion of Radioactive Element into Tracheobronchial Tree, Via Natural or Artificial Opening | ICD-10-PCS |
| 0BH081Z | Insertion of Radioactive Element into Tracheobronchial Tree, Via Natural or Artificial Opening Endoscopic | ICD-10-PCS |
| 0BHK01Z | Insertion of Radioactive Element into Right Lung, Open Approach | ICD-10-PCS |
| 0BHK31Z | Insertion of Radioactive Element into Right Lung, Percutaneous Approach | ICD-10-PCS |
| 0BHK41Z | Insertion of Radioactive Element into Right Lung, Percutaneous Endoscopic Approach | ICD-10-PCS |
| 0BHK71Z | Insertion of Radioactive Element into Right Lung, Via Natural or Artificial Opening | ICD-10-PCS |
| 0BHK81Z | Insertion of Radioactive Element into Right Lung, Via Natural or Artificial Opening Endoscopic | ICD-10-PCS |
| 0BHL01Z | Insertion of Radioactive Element into Left Lung, Open Approach | ICD-10-PCS |
| 0BHL31Z | Insertion of Radioactive Element into Left Lung, Percutaneous Approach | ICD-10-PCS |
| 0BHL41Z | Insertion of Radioactive Element into Left Lung, Percutaneous Endoscopic Approach | ICD-10-PCS |
| 0BHL71Z | Insertion of Radioactive Element into Left Lung, Via Natural or Artificial Opening | ICD-10-PCS |
| 0BHL81Z | Insertion of Radioactive Element into Left Lung, Via Natural or Artificial Opening Endoscopic | ICD-10-PCS |
| 0CH701Z | Insertion of Radioactive Element into Tongue, Open Approach | ICD-10-PCS |
| 0CH731Z | Insertion of Radioactive Element into Tongue, Percutaneous Approach | ICD-10-PCS |
| 0CH7X1Z | Insertion of Radioactive Element into Tongue, External Approach | ICD-10-PCS |
| 0DH501Z | Insertion of Radioactive Element into Esophagus, Open Approach | ICD-10-PCS |
| 0DH531Z | Insertion of Radioactive Element into Esophagus, Percutaneous Approach | ICD-10-PCS |
| 0DH541Z | Insertion of Radioactive Element into Esophagus, Percutaneous Endoscopic Approach | ICD-10-PCS |
| 0DH571Z | Insertion of Radioactive Element into Esophagus, Via Natural or Artificial Opening | ICD-10-PCS |
| 0DH581Z | Insertion of Radioactive Element into Esophagus, Via Natural or Artificial Opening Endoscopic | ICD-10-PCS |
| 0DHP01Z | Insertion of Radioactive Element into Rectum, Open Approach | ICD-10-PCS |
| 0DHP31Z | Insertion of Radioactive Element into Rectum, Percutaneous Approach | ICD-10-PCS |
| 0DHP41Z | Insertion of Radioactive Element into Rectum, Percutaneous Endoscopic Approach | ICD-10-PCS |
| 0DHP71Z | Insertion of Radioactive Element into Rectum, Via Natural or Artificial Opening | ICD-10-PCS |
| 0DHP81Z | Insertion of Radioactive Element into Rectum, Via Natural or Artificial Opening Endoscopic | ICD-10-PCS |
| 0FHB01Z | Insertion of Radioactive Element into Hepatobiliary Duct, Open Approach | ICD-10-PCS |
| 0FHB31Z | Insertion of Radioactive Element into Hepatobiliary Duct, Percutaneous Approach | ICD-10-PCS |
| 0FHB41Z | Insertion of Radioactive Element into Hepatobiliary Duct, Percutaneous Endoscopic Approach | ICD-10-PCS |
| 0FHB71Z | Insertion of Radioactive Element into Hepatobiliary Duct, Via Natural or Artificial Opening | ICD-10-PCS |
| 0FHB81Z | Insertion of Radioactive Element into Hepatobiliary Duct, Via Natural or Artificial Opening Endoscopic | ICD-10-PCS |
| 0FHD01Z | Insertion of Radioactive Element into Pancreatic Duct, Open Approach | ICD-10-PCS |
| 0FHD31Z | Insertion of Radioactive Element into Pancreatic Duct, Percutaneous Approach | ICD-10-PCS |
| 0FHD41Z | Insertion of Radioactive Element into Pancreatic Duct, Percutaneous Endoscopic Approach | ICD-10-PCS |
| 0FHD71Z | Insertion of Radioactive Element into Pancreatic Duct, Via Natural or Artificial Opening | ICD-10-PCS |
| 0FHD81Z | Insertion of Radioactive Element into Pancreatic Duct, Via Natural or Artificial Opening Endoscopic | ICD-10-PCS |
| 0HHT01Z | Insertion of Radioactive Element into Right Breast, Open Approach | ICD-10-PCS |
| 0HHT31Z | Insertion of Radioactive Element into Right Breast, Percutaneous Approach | ICD-10-PCS |
| 0HHT71Z | Insertion of Radioactive Element into Right Breast, Via Natural or Artificial Opening | ICD-10-PCS |
| 0HHT81Z | Insertion of Radioactive Element into Right Breast, Via Natural or Artificial Opening Endoscopic | ICD-10-PCS |
| 0HHTX1Z | Insertion of Radioactive Element into Right Breast, External Approach | ICD-10-PCS |
| 0HHU01Z | Insertion of Radioactive Element into Left Breast, Open Approach | ICD-10-PCS |
| 0HHU31Z | Insertion of Radioactive Element into Left Breast, Percutaneous Approach | ICD-10-PCS |
| 0HHU71Z | Insertion of Radioactive Element into Left Breast, Via Natural or Artificial Opening | ICD-10-PCS |
| 0HHU81Z | Insertion of Radioactive Element into Left Breast, Via Natural or Artificial Opening Endoscopic | ICD-10-PCS |
| 0HHUX1Z | Insertion of Radioactive Element into Left Breast, External Approach | ICD-10-PCS |
| 0HHV01Z | Insertion of Radioactive Element into Bilateral Breast, Open Approach | ICD-10-PCS |
| 0HHV31Z | Insertion of Radioactive Element into Bilateral Breast, Percutaneous Approach | ICD-10-PCS |
| 0HHV71Z | Insertion of Radioactive Element into Bilateral Breast, Via Natural or Artificial Opening | ICD-10-PCS |
| 0HHV81Z | Insertion of Radioactive Element into Bilateral Breast, Via Natural or Artificial Opening Endoscopic | ICD-10-PCS |
| 0HHVX1Z | Insertion of Radioactive Element into Bilateral Breast, External Approach | ICD-10-PCS |
| 0HHW01Z | Insertion of Radioactive Element into Right Nipple, Open Approach | ICD-10-PCS |
| 0HHW31Z | Insertion of Radioactive Element into Right Nipple, Percutaneous Approach | ICD-10-PCS |
| 0HHW71Z | Insertion of Radioactive Element into Right Nipple, Via Natural or Artificial Opening | ICD-10-PCS |
| 0HHW81Z | Insertion of Radioactive Element into Right Nipple, Via Natural or Artificial Opening Endoscopic | ICD-10-PCS |
| 0HHWX1Z | Insertion of Radioactive Element into Right Nipple, External Approach | ICD-10-PCS |
| 0HHX01Z | Insertion of Radioactive Element into Left Nipple, Open Approach | ICD-10-PCS |
| 0HHX31Z | Insertion of Radioactive Element into Left Nipple, Percutaneous Approach | ICD-10-PCS |
| 0HHX71Z | Insertion of Radioactive Element into Left Nipple, Via Natural or Artificial Opening | ICD-10-PCS |
| 0HHX81Z | Insertion of Radioactive Element into Left Nipple, Via Natural or Artificial Opening Endoscopic | ICD-10-PCS |
| 0HHXX1Z | Insertion of Radioactive Element into Left Nipple, External Approach | ICD-10-PCS |
| 0JHS01Z | Insertion of Radioactive Element into Head and Neck Subcutaneous Tissue and Fascia, Open Approach | ICD-10-PCS |
| 0JHS31Z | Insertion of Radioactive Element into Head and Neck Subcutaneous Tissue and Fascia, Percutaneous Approach | ICD-10-PCS |
| 0JHT01Z | Insertion of Radioactive Element into Trunk Subcutaneous Tissue and Fascia, Open Approach | ICD-10-PCS |
| 0JHT31Z | Insertion of Radioactive Element into Trunk Subcutaneous Tissue and Fascia, Percutaneous Approach | ICD-10-PCS |
| 0JHV01Z | Insertion of Radioactive Element into Upper Extremity Subcutaneous Tissue and Fascia, Open Approach | ICD-10-PCS |
| 0JHV31Z | Insertion of Radioactive Element into Upper Extremity Subcutaneous Tissue and Fascia, Percutaneous Approach | ICD-10-PCS |
| 0JHW01Z | Insertion of Radioactive Element into Lower Extremity Subcutaneous Tissue and Fascia, Open Approach | ICD-10-PCS |
| 0JHW31Z | Insertion of Radioactive Element into Lower Extremity Subcutaneous Tissue and Fascia, Percutaneous Approach | ICD-10-PCS |
| 0UHC01Z | Insertion of Radioactive Element into Cervix, Open Approach | ICD-10-PCS |
| 0UHC31Z | Insertion of Radioactive Element into Cervix, Percutaneous Approach | ICD-10-PCS |
| 0UHC41Z | Insertion of Radioactive Element into Cervix, Percutaneous Endoscopic Approach | ICD-10-PCS |
| 0UHC71Z | Insertion of Radioactive Element into Cervix, Via Natural or Artificial Opening | ICD-10-PCS |
| 0UHC81Z | Insertion of Radioactive Element into Cervix, Via Natural or Artificial Opening Endoscopic | ICD-10-PCS |
| 0UHG01Z | Insertion of Radioactive Element into Vagina, Open Approach | ICD-10-PCS |
| 0UHG31Z | Insertion of Radioactive Element into Vagina, Percutaneous Approach | ICD-10-PCS |
| 0UHG41Z | Insertion of Radioactive Element into Vagina, Percutaneous Endoscopic Approach | ICD-10-PCS |
| 0UHG71Z | Insertion of Radioactive Element into Vagina, Via Natural or Artificial Opening | ICD-10-PCS |
| 0UHG81Z | Insertion of Radioactive Element into Vagina, Via Natural or Artificial Opening Endoscopic | ICD-10-PCS |
| 0UHGX1Z | Insertion of Radioactive Element into Vagina, External Approach | ICD-10-PCS |
| 0VH001Z | Insertion of Radioactive Element into Prostate, Open Approach | ICD-10-PCS |
| 0VH031Z | Insertion of Radioactive Element into Prostate, Percutaneous Approach | ICD-10-PCS |
| 0VH041Z | Insertion of Radioactive Element into Prostate, Percutaneous Endoscopic Approach | ICD-10-PCS |
| 0VH071Z | Insertion of Radioactive Element into Prostate, Via Natural or Artificial Opening | ICD-10-PCS |
| 0VH081Z | Insertion of Radioactive Element into Prostate, Via Natural or Artificial Opening Endoscopic | ICD-10-PCS |
| 0WH001Z | Insertion of Radioactive Element into Head, Open Approach | ICD-10-PCS |
| 0WH031Z | Insertion of Radioactive Element into Head, Percutaneous Approach | ICD-10-PCS |
| 0WH041Z | Insertion of Radioactive Element into Head, Percutaneous Endoscopic Approach | ICD-10-PCS |
| 0WH101Z | Insertion of Radioactive Element into Cranial Cavity, Open Approach | ICD-10-PCS |
| 0WH131Z | Insertion of Radioactive Element into Cranial Cavity, Percutaneous Approach | ICD-10-PCS |
| 0WH141Z | Insertion of Radioactive Element into Cranial Cavity, Percutaneous Endoscopic Approach | ICD-10-PCS |
| 0WH201Z | Insertion of Radioactive Element into Face, Open Approach | ICD-10-PCS |
| 0WH231Z | Insertion of Radioactive Element into Face, Percutaneous Approach | ICD-10-PCS |
| 0WH241Z | Insertion of Radioactive Element into Face, Percutaneous Endoscopic Approach | ICD-10-PCS |
| 0WH301Z | Insertion of Radioactive Element into Oral Cavity and Throat, Open Approach | ICD-10-PCS |
| 0WH331Z | Insertion of Radioactive Element into Oral Cavity and Throat, Percutaneous Approach | ICD-10-PCS |
| 0WH341Z | Insertion of Radioactive Element into Oral Cavity and Throat, Percutaneous Endoscopic Approach | ICD-10-PCS |
| 0WH401Z | Insertion of Radioactive Element into Upper Jaw, Open Approach | ICD-10-PCS |
| 0WH431Z | Insertion of Radioactive Element into Upper Jaw, Percutaneous Approach | ICD-10-PCS |
| 0WH441Z | Insertion of Radioactive Element into Upper Jaw, Percutaneous Endoscopic Approach | ICD-10-PCS |
| 0WH501Z | Insertion of Radioactive Element into Lower Jaw, Open Approach | ICD-10-PCS |
| 0WH531Z | Insertion of Radioactive Element into Lower Jaw, Percutaneous Approach | ICD-10-PCS |
| 0WH541Z | Insertion of Radioactive Element into Lower Jaw, Percutaneous Endoscopic Approach | ICD-10-PCS |
| 0WH601Z | Insertion of Radioactive Element into Neck, Open Approach | ICD-10-PCS |
| 0WH631Z | Insertion of Radioactive Element into Neck, Percutaneous Approach | ICD-10-PCS |
| 0WH641Z | Insertion of Radioactive Element into Neck, Percutaneous Endoscopic Approach | ICD-10-PCS |
| 0WH801Z | Insertion of Radioactive Element into Chest Wall, Open Approach | ICD-10-PCS |
| 0WH831Z | Insertion of Radioactive Element into Chest Wall, Percutaneous Approach | ICD-10-PCS |
| 0WH841Z | Insertion of Radioactive Element into Chest Wall, Percutaneous Endoscopic Approach | ICD-10-PCS |
| 0WH901Z | Insertion of Radioactive Element into Right Pleural Cavity, Open Approach | ICD-10-PCS |
| 0WH931Z | Insertion of Radioactive Element into Right Pleural Cavity, Percutaneous Approach | ICD-10-PCS |
| 0WH941Z | Insertion of Radioactive Element into Right Pleural Cavity, Percutaneous Endoscopic Approach | ICD-10-PCS |
| 0WHB01Z | Insertion of Radioactive Element into Left Pleural Cavity, Open Approach | ICD-10-PCS |
| 0WHB31Z | Insertion of Radioactive Element into Left Pleural Cavity, Percutaneous Approach | ICD-10-PCS |
| 0WHB41Z | Insertion of Radioactive Element into Left Pleural Cavity, Percutaneous Endoscopic Approach | ICD-10-PCS |
| 0WHC01Z | Insertion of Radioactive Element into Mediastinum, Open Approach | ICD-10-PCS |
| 0WHC31Z | Insertion of Radioactive Element into Mediastinum, Percutaneous Approach | ICD-10-PCS |
| 0WHC41Z | Insertion of Radioactive Element into Mediastinum, Percutaneous Endoscopic Approach | ICD-10-PCS |
| 0WHD01Z | Insertion of Radioactive Element into Pericardial Cavity, Open Approach | ICD-10-PCS |
| 0WHD31Z | Insertion of Radioactive Element into Pericardial Cavity, Percutaneous Approach | ICD-10-PCS |
| 0WHD41Z | Insertion of Radioactive Element into Pericardial Cavity, Percutaneous Endoscopic Approach | ICD-10-PCS |
| 0WHF01Z | Insertion of Radioactive Element into Abdominal Wall, Open Approach | ICD-10-PCS |
| 0WHF31Z | Insertion of Radioactive Element into Abdominal Wall, Percutaneous Approach | ICD-10-PCS |
| 0WHF41Z | Insertion of Radioactive Element into Abdominal Wall, Percutaneous Endoscopic Approach | ICD-10-PCS |
| 0WHG01Z | Insertion of Radioactive Element into Peritoneal Cavity, Open Approach | ICD-10-PCS |
| 0WHG31Z | Insertion of Radioactive Element into Peritoneal Cavity, Percutaneous Approach | ICD-10-PCS |
| 0WHG41Z | Insertion of Radioactive Element into Peritoneal Cavity, Percutaneous Endoscopic Approach | ICD-10-PCS |
| 0WHH01Z | Insertion of Radioactive Element into Retroperitoneum, Open Approach | ICD-10-PCS |
| 0WHH31Z | Insertion of Radioactive Element into Retroperitoneum, Percutaneous Approach | ICD-10-PCS |
| 0WHH41Z | Insertion of Radioactive Element into Retroperitoneum, Percutaneous Endoscopic Approach | ICD-10-PCS |
| 0WHJ01Z | Insertion of Radioactive Element into Pelvic Cavity, Open Approach | ICD-10-PCS |
| 0WHJ31Z | Insertion of Radioactive Element into Pelvic Cavity, Percutaneous Approach | ICD-10-PCS |
| 0WHJ41Z | Insertion of Radioactive Element into Pelvic Cavity, Percutaneous Endoscopic Approach | ICD-10-PCS |
| 0WHK01Z | Insertion of Radioactive Element into Upper Back, Open Approach | ICD-10-PCS |
| 0WHK31Z | Insertion of Radioactive Element into Upper Back, Percutaneous Approach | ICD-10-PCS |
| 0WHK41Z | Insertion of Radioactive Element into Upper Back, Percutaneous Endoscopic Approach | ICD-10-PCS |
| 0WHL01Z | Insertion of Radioactive Element into Lower Back, Open Approach | ICD-10-PCS |
| 0WHL31Z | Insertion of Radioactive Element into Lower Back, Percutaneous Approach | ICD-10-PCS |
| 0WHL41Z | Insertion of Radioactive Element into Lower Back, Percutaneous Endoscopic Approach | ICD-10-PCS |
| 0WHM01Z | Insertion of Radioactive Element into Male Perineum, Open Approach | ICD-10-PCS |
| 0WHM31Z | Insertion of Radioactive Element into Male Perineum, Percutaneous Approach | ICD-10-PCS |
| 0WHM41Z | Insertion of Radioactive Element into Male Perineum, Percutaneous Endoscopic Approach | ICD-10-PCS |
| 0WHN01Z | Insertion of Radioactive Element into Female Perineum, Open Approach | ICD-10-PCS |
| 0WHN31Z | Insertion of Radioactive Element into Female Perineum, Percutaneous Approach | ICD-10-PCS |
| 0WHN41Z | Insertion of Radioactive Element into Female Perineum, Percutaneous Endoscopic Approach | ICD-10-PCS |
| 0WHP01Z | Insertion of Radioactive Element into Gastrointestinal Tract, Open Approach | ICD-10-PCS |
| 0WHP31Z | Insertion of Radioactive Element into Gastrointestinal Tract, Percutaneous Approach | ICD-10-PCS |
| 0WHP41Z | Insertion of Radioactive Element into Gastrointestinal Tract, Percutaneous Endoscopic Approach | ICD-10-PCS |
| 0WHP71Z | Insertion of Radioactive Element into Gastrointestinal Tract, Via Natural or Artificial Opening | ICD-10-PCS |
| 0WHP81Z | Insertion of Radioactive Element into Gastrointestinal Tract, Via Natural or Artificial Opening Endoscopic | ICD-10-PCS |
| 0WHQ01Z | Insertion of Radioactive Element into Respiratory Tract, Open Approach | ICD-10-PCS |
| 0WHQ31Z | Insertion of Radioactive Element into Respiratory Tract, Percutaneous Approach | ICD-10-PCS |
| 0WHQ41Z | Insertion of Radioactive Element into Respiratory Tract, Percutaneous Endoscopic Approach | ICD-10-PCS |
| 0WHQ71Z | Insertion of Radioactive Element into Respiratory Tract, Via Natural or Artificial Opening | ICD-10-PCS |
| 0WHQ81Z | Insertion of Radioactive Element into Respiratory Tract, Via Natural or Artificial Opening Endoscopic | ICD-10-PCS |
| 0WHR01Z | Insertion of Radioactive Element into Genitourinary Tract, Open Approach | ICD-10-PCS |
| 0WHR31Z | Insertion of Radioactive Element into Genitourinary Tract, Percutaneous Approach | ICD-10-PCS |
| 0WHR41Z | Insertion of Radioactive Element into Genitourinary Tract, Percutaneous Endoscopic Approach | ICD-10-PCS |
| 0WHR71Z | Insertion of Radioactive Element into Genitourinary Tract, Via Natural or Artificial Opening | ICD-10-PCS |
| 0WHR81Z | Insertion of Radioactive Element into Genitourinary Tract, Via Natural or Artificial Opening Endoscopic | ICD-10-PCS |
| 0XH201Z | Insertion of Radioactive Element into Right Shoulder Region, Open Approach | ICD-10-PCS |
| 0XH231Z | Insertion of Radioactive Element into Right Shoulder Region, Percutaneous Approach | ICD-10-PCS |
| 0XH241Z | Insertion of Radioactive Element into Right Shoulder Region, Percutaneous Endoscopic Approach | ICD-10-PCS |
| 0XH301Z | Insertion of Radioactive Element into Left Shoulder Region, Open Approach | ICD-10-PCS |
| 0XH331Z | Insertion of Radioactive Element into Left Shoulder Region, Percutaneous Approach | ICD-10-PCS |
| 0XH341Z | Insertion of Radioactive Element into Left Shoulder Region, Percutaneous Endoscopic Approach | ICD-10-PCS |
| 0XH401Z | Insertion of Radioactive Element into Right Axilla, Open Approach | ICD-10-PCS |
| 0XH431Z | Insertion of Radioactive Element into Right Axilla, Percutaneous Approach | ICD-10-PCS |
| 0XH441Z | Insertion of Radioactive Element into Right Axilla, Percutaneous Endoscopic Approach | ICD-10-PCS |
| 0XH501Z | Insertion of Radioactive Element into Left Axilla, Open Approach | ICD-10-PCS |
| 0XH531Z | Insertion of Radioactive Element into Left Axilla, Percutaneous Approach | ICD-10-PCS |
| 0XH541Z | Insertion of Radioactive Element into Left Axilla, Percutaneous Endoscopic Approach | ICD-10-PCS |
| 0XH601Z | Insertion of Radioactive Element into Right Upper Extremity, Open Approach | ICD-10-PCS |
| 0XH631Z | Insertion of Radioactive Element into Right Upper Extremity, Percutaneous Approach | ICD-10-PCS |
| 0XH641Z | Insertion of Radioactive Element into Right Upper Extremity, Percutaneous Endoscopic Approach | ICD-10-PCS |
| 0XH701Z | Insertion of Radioactive Element into Left Upper Extremity, Open Approach | ICD-10-PCS |
| 0XH731Z | Insertion of Radioactive Element into Left Upper Extremity, Percutaneous Approach | ICD-10-PCS |
| 0XH741Z | Insertion of Radioactive Element into Left Upper Extremity, Percutaneous Endoscopic Approach | ICD-10-PCS |
| 0XH801Z | Insertion of Radioactive Element into Right Upper Arm, Open Approach | ICD-10-PCS |
| 0XH831Z | Insertion of Radioactive Element into Right Upper Arm, Percutaneous Approach | ICD-10-PCS |
| 0XH841Z | Insertion of Radioactive Element into Right Upper Arm, Percutaneous Endoscopic Approach | ICD-10-PCS |
| 0XH901Z | Insertion of Radioactive Element into Left Upper Arm, Open Approach | ICD-10-PCS |
| 0XH931Z | Insertion of Radioactive Element into Left Upper Arm, Percutaneous Approach | ICD-10-PCS |
| 0XH941Z | Insertion of Radioactive Element into Left Upper Arm, Percutaneous Endoscopic Approach | ICD-10-PCS |
| 0XHB01Z | Insertion of Radioactive Element into Right Elbow Region, Open Approach | ICD-10-PCS |
| 0XHB31Z | Insertion of Radioactive Element into Right Elbow Region, Percutaneous Approach | ICD-10-PCS |
| 0XHB41Z | Insertion of Radioactive Element into Right Elbow Region, Percutaneous Endoscopic Approach | ICD-10-PCS |
| 0XHC01Z | Insertion of Radioactive Element into Left Elbow Region, Open Approach | ICD-10-PCS |
| 0XHC31Z | Insertion of Radioactive Element into Left Elbow Region, Percutaneous Approach | ICD-10-PCS |
| 0XHC41Z | Insertion of Radioactive Element into Left Elbow Region, Percutaneous Endoscopic Approach | ICD-10-PCS |
| 0XHD01Z | Insertion of Radioactive Element into Right Lower Arm, Open Approach | ICD-10-PCS |
| 0XHD31Z | Insertion of Radioactive Element into Right Lower Arm, Percutaneous Approach | ICD-10-PCS |
| 0XHD41Z | Insertion of Radioactive Element into Right Lower Arm, Percutaneous Endoscopic Approach | ICD-10-PCS |
| 0XHF01Z | Insertion of Radioactive Element into Left Lower Arm, Open Approach | ICD-10-PCS |
| 0XHF31Z | Insertion of Radioactive Element into Left Lower Arm, Percutaneous Approach | ICD-10-PCS |
| 0XHF41Z | Insertion of Radioactive Element into Left Lower Arm, Percutaneous Endoscopic Approach | ICD-10-PCS |
| 0XHG01Z | Insertion of Radioactive Element into Right Wrist Region, Open Approach | ICD-10-PCS |
| 0XHG31Z | Insertion of Radioactive Element into Right Wrist Region, Percutaneous Approach | ICD-10-PCS |
| 0XHG41Z | Insertion of Radioactive Element into Right Wrist Region, Percutaneous Endoscopic Approach | ICD-10-PCS |
| 0XHH01Z | Insertion of Radioactive Element into Left Wrist Region, Open Approach | ICD-10-PCS |
| 0XHH31Z | Insertion of Radioactive Element into Left Wrist Region, Percutaneous Approach | ICD-10-PCS |
| 0XHH41Z | Insertion of Radioactive Element into Left Wrist Region, Percutaneous Endoscopic Approach | ICD-10-PCS |
| 0XHJ01Z | Insertion of Radioactive Element into Right Hand, Open Approach | ICD-10-PCS |
| 0XHJ31Z | Insertion of Radioactive Element into Right Hand, Percutaneous Approach | ICD-10-PCS |
| 0XHJ41Z | Insertion of Radioactive Element into Right Hand, Percutaneous Endoscopic Approach | ICD-10-PCS |
| 0XHK01Z | Insertion of Radioactive Element into Left Hand, Open Approach | ICD-10-PCS |
| 0XHK31Z | Insertion of Radioactive Element into Left Hand, Percutaneous Approach | ICD-10-PCS |
| 0XHK41Z | Insertion of Radioactive Element into Left Hand, Percutaneous Endoscopic Approach | ICD-10-PCS |
| 0YH001Z | Insertion of Radioactive Element into Right Buttock, Open Approach | ICD-10-PCS |
| 0YH031Z | Insertion of Radioactive Element into Right Buttock, Percutaneous Approach | ICD-10-PCS |
| 0YH041Z | Insertion of Radioactive Element into Right Buttock, Percutaneous Endoscopic Approach | ICD-10-PCS |
| 0YH101Z | Insertion of Radioactive Element into Left Buttock, Open Approach | ICD-10-PCS |
| 0YH131Z | Insertion of Radioactive Element into Left Buttock, Percutaneous Approach | ICD-10-PCS |
| 0YH141Z | Insertion of Radioactive Element into Left Buttock, Percutaneous Endoscopic Approach | ICD-10-PCS |
| 0YH501Z | Insertion of Radioactive Element into Right Inguinal Region, Open Approach | ICD-10-PCS |
| 0YH531Z | Insertion of Radioactive Element into Right Inguinal Region, Percutaneous Approach | ICD-10-PCS |
| 0YH541Z | Insertion of Radioactive Element into Right Inguinal Region, Percutaneous Endoscopic Approach | ICD-10-PCS |
| 0YH601Z | Insertion of Radioactive Element into Left Inguinal Region, Open Approach | ICD-10-PCS |
| 0YH631Z | Insertion of Radioactive Element into Left Inguinal Region, Percutaneous Approach | ICD-10-PCS |
| 0YH641Z | Insertion of Radioactive Element into Left Inguinal Region, Percutaneous Endoscopic Approach | ICD-10-PCS |
| 0YH701Z | Insertion of Radioactive Element into Right Femoral Region, Open Approach | ICD-10-PCS |
| 0YH731Z | Insertion of Radioactive Element into Right Femoral Region, Percutaneous Approach | ICD-10-PCS |
| 0YH741Z | Insertion of Radioactive Element into Right Femoral Region, Percutaneous Endoscopic Approach | ICD-10-PCS |
| 0YH801Z | Insertion of Radioactive Element into Left Femoral Region, Open Approach | ICD-10-PCS |
| 0YH831Z | Insertion of Radioactive Element into Left Femoral Region, Percutaneous Approach | ICD-10-PCS |
| 0YH841Z | Insertion of Radioactive Element into Left Femoral Region, Percutaneous Endoscopic Approach | ICD-10-PCS |
| 0YH901Z | Insertion of Radioactive Element into Right Lower Extremity, Open Approach | ICD-10-PCS |
| 0YH931Z | Insertion of Radioactive Element into Right Lower Extremity, Percutaneous Approach | ICD-10-PCS |
| 0YH941Z | Insertion of Radioactive Element into Right Lower Extremity, Percutaneous Endoscopic Approach | ICD-10-PCS |
| 0YHB01Z | Insertion of Radioactive Element into Left Lower Extremity, Open Approach | ICD-10-PCS |
| 0YHB31Z | Insertion of Radioactive Element into Left Lower Extremity, Percutaneous Approach | ICD-10-PCS |
| 0YHB41Z | Insertion of Radioactive Element into Left Lower Extremity, Percutaneous Endoscopic Approach | ICD-10-PCS |
| 0YHC01Z | Insertion of Radioactive Element into Right Upper Leg, Open Approach | ICD-10-PCS |
| 0YHC31Z | Insertion of Radioactive Element into Right Upper Leg, Percutaneous Approach | ICD-10-PCS |
| 0YHC41Z | Insertion of Radioactive Element into Right Upper Leg, Percutaneous Endoscopic Approach | ICD-10-PCS |
| 0YHD01Z | Insertion of Radioactive Element into Left Upper Leg, Open Approach | ICD-10-PCS |
| 0YHD31Z | Insertion of Radioactive Element into Left Upper Leg, Percutaneous Approach | ICD-10-PCS |
| 0YHD41Z | Insertion of Radioactive Element into Left Upper Leg, Percutaneous Endoscopic Approach | ICD-10-PCS |
| 0YHF01Z | Insertion of Radioactive Element into Right Knee Region, Open Approach | ICD-10-PCS |
| 0YHF31Z | Insertion of Radioactive Element into Right Knee Region, Percutaneous Approach | ICD-10-PCS |
| 0YHF41Z | Insertion of Radioactive Element into Right Knee Region, Percutaneous Endoscopic Approach | ICD-10-PCS |
| 0YHG01Z | Insertion of Radioactive Element into Left Knee Region, Open Approach | ICD-10-PCS |
| 0YHG31Z | Insertion of Radioactive Element into Left Knee Region, Percutaneous Approach | ICD-10-PCS |
| 0YHG41Z | Insertion of Radioactive Element into Left Knee Region, Percutaneous Endoscopic Approach | ICD-10-PCS |
| 0YHH01Z | Insertion of Radioactive Element into Right Lower Leg, Open Approach | ICD-10-PCS |
| 0YHH31Z | Insertion of Radioactive Element into Right Lower Leg, Percutaneous Approach | ICD-10-PCS |
| 0YHH41Z | Insertion of Radioactive Element into Right Lower Leg, Percutaneous Endoscopic Approach | ICD-10-PCS |
| 0YHJ01Z | Insertion of Radioactive Element into Left Lower Leg, Open Approach | ICD-10-PCS |
| 0YHJ31Z | Insertion of Radioactive Element into Left Lower Leg, Percutaneous Approach | ICD-10-PCS |
| 0YHJ41Z | Insertion of Radioactive Element into Left Lower Leg, Percutaneous Endoscopic Approach | ICD-10-PCS |
| 0YHK01Z | Insertion of Radioactive Element into Right Ankle Region, Open Approach | ICD-10-PCS |
| 0YHK31Z | Insertion of Radioactive Element into Right Ankle Region, Percutaneous Approach | ICD-10-PCS |
| 0YHK41Z | Insertion of Radioactive Element into Right Ankle Region, Percutaneous Endoscopic Approach | ICD-10-PCS |
| 0YHL01Z | Insertion of Radioactive Element into Left Ankle Region, Open Approach | ICD-10-PCS |
| 0YHL31Z | Insertion of Radioactive Element into Left Ankle Region, Percutaneous Approach | ICD-10-PCS |
| 0YHL41Z | Insertion of Radioactive Element into Left Ankle Region, Percutaneous Endoscopic Approach | ICD-10-PCS |
| 0YHM01Z | Insertion of Radioactive Element into Right Foot, Open Approach | ICD-10-PCS |
| 0YHM31Z | Insertion of Radioactive Element into Right Foot, Percutaneous Approach | ICD-10-PCS |
| 0YHM41Z | Insertion of Radioactive Element into Right Foot, Percutaneous Endoscopic Approach | ICD-10-PCS |
| 0YHN01Z | Insertion of Radioactive Element into Left Foot, Open Approach | ICD-10-PCS |
| 0YHN31Z | Insertion of Radioactive Element into Left Foot, Percutaneous Approach | ICD-10-PCS |
| 0YHN41Z | Insertion of Radioactive Element into Left Foot, Percutaneous Endoscopic Approach | ICD-10-PCS |
| 3E0B304 | Introduction of Liquid Brachytherapy Radioisotope into Ear, Percutaneous Approach | ICD-10-PCS |
| 3E0B704 | Introduction of Liquid Brachytherapy Radioisotope into Ear, Via Natural or Artificial Opening | ICD-10-PCS |
| 3E0BX04 | Introduction of Liquid Brachytherapy Radioisotope into Ear, External Approach | ICD-10-PCS |
| 3E0C304 | Introduction of Liquid Brachytherapy Radioisotope into Eye, Percutaneous Approach | ICD-10-PCS |
| 3E0C704 | Introduction of Liquid Brachytherapy Radioisotope into Eye, Via Natural or Artificial Opening | ICD-10-PCS |
| 3E0CX04 | Introduction of Liquid Brachytherapy Radioisotope into Eye, External Approach | ICD-10-PCS |
| 3E0D304 | Introduction of Liquid Brachytherapy Radioisotope into Mouth and Pharynx, Percutaneous Approach | ICD-10-PCS |
| 3E0D704 | Introduction of Liquid Brachytherapy Radioisotope into Mouth and Pharynx, Via Natural or Artificial Opening | ICD-10-PCS |
| 3E0DX04 | Introduction of Liquid Brachytherapy Radioisotope into Mouth and Pharynx, External Approach | ICD-10-PCS |
| 3E0E304 | Introduction of Liquid Brachytherapy Radioisotope into Products of Conception, Percutaneous Approach | ICD-10-PCS |
| 3E0E704 | Introduction of Liquid Brachytherapy Radioisotope into Products of Conception, Via Natural or Artificial Opening | ICD-10-PCS |
| 3E0E804 | Introduction of Liquid Brachytherapy Radioisotope into Products of Conception, Via Natural or Artificial Opening Endoscopic | ICD-10-PCS |
| 3E0F304 | Introduction of Liquid Brachytherapy Radioisotope into Respiratory Tract, Percutaneous Approach | ICD-10-PCS |
| 3E0F704 | Introduction of Liquid Brachytherapy Radioisotope into Respiratory Tract, Via Natural or Artificial Opening | ICD-10-PCS |
| 3E0F804 | Introduction of Liquid Brachytherapy Radioisotope into Respiratory Tract, Via Natural or Artificial Opening Endoscopic | ICD-10-PCS |
| 3E0G304 | Introduction of Liquid Brachytherapy Radioisotope into Upper GI, Percutaneous Approach | ICD-10-PCS |
| 3E0G704 | Introduction of Liquid Brachytherapy Radioisotope into Upper GI, Via Natural or Artificial Opening | ICD-10-PCS |
| 3E0G804 | Introduction of Liquid Brachytherapy Radioisotope into Upper GI, Via Natural or Artificial Opening Endoscopic | ICD-10-PCS |
| 3E0H304 | Introduction of Liquid Brachytherapy Radioisotope into Lower GI, Percutaneous Approach | ICD-10-PCS |
| 3E0H704 | Introduction of Liquid Brachytherapy Radioisotope into Lower GI, Via Natural or Artificial Opening | ICD-10-PCS |
| 3E0H804 | Introduction of Liquid Brachytherapy Radioisotope into Lower GI, Via Natural or Artificial Opening Endoscopic | ICD-10-PCS |
| 3E0J304 | Introduction of Liquid Brachytherapy Radioisotope into Biliary and Pancreatic Tract, Percutaneous Approach | ICD-10-PCS |
| 3E0J704 | Introduction of Liquid Brachytherapy Radioisotope into Biliary and Pancreatic Tract, Via Natural or Artificial Opening | ICD-10-PCS |
| 3E0J804 | Introduction of Liquid Brachytherapy Radioisotope into Biliary and Pancreatic Tract, Via Natural or Artificial Opening Endoscopic | ICD-10-PCS |
| 3E0K304 | Introduction of Liquid Brachytherapy Radioisotope into Genitourinary Tract, Percutaneous Approach | ICD-10-PCS |
| 3E0K704 | Introduction of Liquid Brachytherapy Radioisotope into Genitourinary Tract, Via Natural or Artificial Opening | ICD-10-PCS |
| 3E0K804 | Introduction of Liquid Brachytherapy Radioisotope into Genitourinary Tract, Via Natural or Artificial Opening Endoscopic | ICD-10-PCS |
| 3E0L304 | Introduction of Liquid Brachytherapy Radioisotope into Pleural Cavity, Percutaneous Approach | ICD-10-PCS |
| 3E0L704 | Introduction of Liquid Brachytherapy Radioisotope into Pleural Cavity, Via Natural or Artificial Opening | ICD-10-PCS |
| 3E0M304 | Introduction of Liquid Brachytherapy Radioisotope into Peritoneal Cavity, Percutaneous Approach | ICD-10-PCS |
| 3E0M704 | Introduction of Liquid Brachytherapy Radioisotope into Peritoneal Cavity, Via Natural or Artificial Opening | ICD-10-PCS |
| 3E0N304 | Introduction of Liquid Brachytherapy Radioisotope into Male Reproductive, Percutaneous Approach | ICD-10-PCS |
| 3E0N704 | Introduction of Liquid Brachytherapy Radioisotope into Male Reproductive, Via Natural or Artificial Opening | ICD-10-PCS |
| 3E0N804 | Introduction of Liquid Brachytherapy Radioisotope into Male Reproductive, Via Natural or Artificial Opening Endoscopic | ICD-10-PCS |
| 3E0P304 | Introduction of Liquid Brachytherapy Radioisotope into Female Reproductive, Percutaneous Approach | ICD-10-PCS |
| 3E0P704 | Introduction of Liquid Brachytherapy Radioisotope into Female Reproductive, Via Natural or Artificial Opening | ICD-10-PCS |
| 3E0P804 | Introduction of Liquid Brachytherapy Radioisotope into Female Reproductive, Via Natural or Artificial Opening Endoscopic | ICD-10-PCS |
| 3E0Q304 | Introduction of Liquid Brachytherapy Radioisotope into Cranial Cavity and Brain, Percutaneous Approach | ICD-10-PCS |
| 3E0Q704 | Introduction of Liquid Brachytherapy Radioisotope into Cranial Cavity and Brain, Via Natural or Artificial Opening | ICD-10-PCS |
| 3E0R304 | Introduction of Liquid Brachytherapy Radioisotope into Spinal Canal, Percutaneous Approach | ICD-10-PCS |
| 3E0S304 | Introduction of Liquid Brachytherapy Radioisotope into Epidural Space, Percutaneous Approach | ICD-10-PCS |
| 3E0U304 | Introduction of Liquid Brachytherapy Radioisotope into Joints, Percutaneous Approach | ICD-10-PCS |
| 3E0Y304 | Introduction of Liquid Brachytherapy Radioisotope into Pericardial Cavity, Percutaneous Approach | ICD-10-PCS |
| 3E0Y704 | Introduction of Liquid Brachytherapy Radioisotope into Pericardial Cavity, Via Natural or Artificial Opening | ICD-10-PCS |
| CW7 | Nuclear Medicine, Anatomical Regions, Systemic Nuclear Medicine Therapy | ICD-10-PCS |
| D00 | Radiation Therapy, Central and Peripheral Nervous System, Beam Radiation | ICD-10-PCS |
| D01 | Radiation Therapy, Central and Peripheral Nervous System, Brachytherapy | ICD-10-PCS |
| D02 | Radiation Therapy, Central and Peripheral Nervous System, Stereotactic Radiosurgery | ICD-10-PCS |
| D0Y07ZZ | Contact Radiation of Brain | ICD-10-PCS |
| D0Y0FZZ | Plaque Radiation of Brain | ICD-10-PCS |
| D0Y17ZZ | Contact Radiation of Brain Stem | ICD-10-PCS |
| D0Y1FZZ | Plaque Radiation of Brain Stem | ICD-10-PCS |
| D0Y67ZZ | Contact Radiation of Spinal Cord | ICD-10-PCS |
| D0Y6FZZ | Plaque Radiation of Spinal Cord | ICD-10-PCS |
| D0Y77ZZ | Contact Radiation of Peripheral Nerve | ICD-10-PCS |
| D0Y7FZZ | Plaque Radiation of Peripheral Nerve | ICD-10-PCS |
| D70 | Radiation Therapy, Lymphatic and Hematologic System, Beam Radiation | ICD-10-PCS |
| D71 | Radiation Therapy, Lymphatic and Hematologic System, Brachytherapy | ICD-10-PCS |
| D72 | Radiation Therapy, Lymphatic and Hematologic System, Stereotactic Radiosurgery | ICD-10-PCS |
| D7Y0FZZ | Plaque Radiation of Bone Marrow | ICD-10-PCS |
| D7Y1FZZ | Plaque Radiation of Thymus | ICD-10-PCS |
| D7Y2FZZ | Plaque Radiation of Spleen | ICD-10-PCS |
| D7Y3FZZ | Plaque Radiation of Neck Lymphatics | ICD-10-PCS |
| D7Y4FZZ | Plaque Radiation of Axillary Lymphatics | ICD-10-PCS |
| D7Y5FZZ | Plaque Radiation of Thorax Lymphatics | ICD-10-PCS |
| D7Y6FZZ | Plaque Radiation of Abdomen Lymphatics | ICD-10-PCS |
| D7Y7FZZ | Plaque Radiation of Pelvis Lymphatics | ICD-10-PCS |
| D7Y8FZZ | Plaque Radiation of Inguinal Lymphatics | ICD-10-PCS |
| D80 | Radiation Therapy, Eye, Beam Radiation | ICD-10-PCS |
| D81 | Radiation Therapy, Eye, Brachytherapy | ICD-10-PCS |
| D82 | Radiation Therapy, Eye, Stereotactic Radiosurgery | ICD-10-PCS |
| D8Y07ZZ | Contact Radiation of Eye | ICD-10-PCS |
| D8Y0FZZ | Plaque Radiation of Eye | ICD-10-PCS |
| D90 | Radiation Therapy, Ear, Nose, Mouth and Throat, Beam Radiation | ICD-10-PCS |
| D91 | Radiation Therapy, Ear, Nose, Mouth and Throat, Brachytherapy | ICD-10-PCS |
| D92 | Radiation Therapy, Ear, Nose, Mouth and Throat, Stereotactic Radiosurgery | ICD-10-PCS |
| D9Y07ZZ | Contact Radiation of Ear | ICD-10-PCS |
| D9Y0FZZ | Plaque Radiation of Ear | ICD-10-PCS |
| D9Y17ZZ | Contact Radiation of Nose | ICD-10-PCS |
| D9Y1FZZ | Plaque Radiation of Nose | ICD-10-PCS |
| D9Y37ZZ | Contact Radiation of Hypopharynx | ICD-10-PCS |
| D9Y47ZZ | Contact Radiation of Mouth | ICD-10-PCS |
| D9Y4CZZ | Intraoperative Radiation Therapy (IORT) of Mouth | ICD-10-PCS |
| D9Y4FZZ | Plaque Radiation of Mouth | ICD-10-PCS |
| D9Y57ZZ | Contact Radiation of Tongue | ICD-10-PCS |
| D9Y5FZZ | Plaque Radiation of Tongue | ICD-10-PCS |
| D9Y67ZZ | Contact Radiation of Salivary Glands | ICD-10-PCS |
| D9Y6FZZ | Plaque Radiation of Salivary Glands | ICD-10-PCS |
| D9Y77ZZ | Contact Radiation of Sinuses | ICD-10-PCS |
| D9Y7FZZ | Plaque Radiation of Sinuses | ICD-10-PCS |
| D9Y87ZZ | Contact Radiation of Hard Palate | ICD-10-PCS |
| D9Y8FZZ | Plaque Radiation of Hard Palate | ICD-10-PCS |
| D9Y97ZZ | Contact Radiation of Soft Palate | ICD-10-PCS |
| D9Y9FZZ | Plaque Radiation of Soft Palate | ICD-10-PCS |
| D9YB7ZZ | Contact Radiation of Larynx | ICD-10-PCS |
| D9YBCZZ | Intraoperative Radiation Therapy (IORT) of Larynx | ICD-10-PCS |
| D9YBFZZ | Plaque Radiation of Larynx | ICD-10-PCS |
| D9YCCZZ | Intraoperative Radiation Therapy (IORT) of Pharynx | ICD-10-PCS |
| D9YCFZZ | Plaque Radiation of Pharynx | ICD-10-PCS |
| D9YD7ZZ | Contact Radiation of Nasopharynx | ICD-10-PCS |
| D9YDCZZ | Intraoperative Radiation Therapy (IORT) of Nasopharynx | ICD-10-PCS |
| D9YDFZZ | Plaque Radiation of Nasopharynx | ICD-10-PCS |
| D9YF7ZZ | Contact Radiation of Oropharynx | ICD-10-PCS |
| DB0 | Radiation Therapy, Respiratory System, Beam Radiation | ICD-10-PCS |
| DB1 | Radiation Therapy, Respiratory System, Brachytherapy | ICD-10-PCS |
| DB2 | Radiation Therapy, Respiratory System, Stereotactic Radiosurgery | ICD-10-PCS |
| DBY07ZZ | Contact Radiation of Trachea | ICD-10-PCS |
| DBY0FZZ | Plaque Radiation of Trachea | ICD-10-PCS |
| DBY17ZZ | Contact Radiation of Bronchus | ICD-10-PCS |
| DBY1FZZ | Plaque Radiation of Bronchus | ICD-10-PCS |
| DBY27ZZ | Contact Radiation of Lung | ICD-10-PCS |
| DBY2FZZ | Plaque Radiation of Lung | ICD-10-PCS |
| DBY57ZZ | Contact Radiation of Pleura | ICD-10-PCS |
| DBY5FZZ | Plaque Radiation of Pleura | ICD-10-PCS |
| DBY67ZZ | Contact Radiation of Mediastinum | ICD-10-PCS |
| DBY6FZZ | Plaque Radiation of Mediastinum | ICD-10-PCS |
| DBY77ZZ | Contact Radiation of Chest Wall | ICD-10-PCS |
| DBY7FZZ | Plaque Radiation of Chest Wall | ICD-10-PCS |
| DBY87ZZ | Contact Radiation of Diaphragm | ICD-10-PCS |
| DBY8FZZ | Plaque Radiation of Diaphragm | ICD-10-PCS |
| DD0 | Radiation Therapy, Gastrointestinal System, Beam Radiation | ICD-10-PCS |
| DD1 | Radiation Therapy, Gastrointestinal System, Brachytherapy | ICD-10-PCS |
| DD2 | Radiation Therapy, Gastrointestinal System, Stereotactic Radiosurgery | ICD-10-PCS |
| DDY07ZZ | Contact Radiation of Esophagus | ICD-10-PCS |
| DDY0FZZ | Plaque Radiation of Esophagus | ICD-10-PCS |
| DDY17ZZ | Contact Radiation of Stomach | ICD-10-PCS |
| DDY1CZZ | Intraoperative Radiation Therapy (IORT) of Stomach | ICD-10-PCS |
| DDY1FZZ | Plaque Radiation of Stomach | ICD-10-PCS |
| DDY27ZZ | Contact Radiation of Duodenum | ICD-10-PCS |
| DDY2CZZ | Intraoperative Radiation Therapy (IORT) of Duodenum | ICD-10-PCS |
| DDY2FZZ | Plaque Radiation of Duodenum | ICD-10-PCS |
| DDY37ZZ | Contact Radiation of Jejunum | ICD-10-PCS |
| DDY3CZZ | Intraoperative Radiation Therapy (IORT) of Jejunum | ICD-10-PCS |
| DDY3FZZ | Plaque Radiation of Jejunum | ICD-10-PCS |
| DDY47ZZ | Contact Radiation of Ileum | ICD-10-PCS |
| DDY4CZZ | Intraoperative Radiation Therapy (IORT) of Ileum | ICD-10-PCS |
| DDY4FZZ | Plaque Radiation of Ileum | ICD-10-PCS |
| DDY57ZZ | Contact Radiation of Colon | ICD-10-PCS |
| DDY5CZZ | Intraoperative Radiation Therapy (IORT) of Colon | ICD-10-PCS |
| DDY5FZZ | Plaque Radiation of Colon | ICD-10-PCS |
| DDY77ZZ | Contact Radiation of Rectum | ICD-10-PCS |
| DDY7CZZ | Intraoperative Radiation Therapy (IORT) of Rectum | ICD-10-PCS |
| DDY7FZZ | Plaque Radiation of Rectum | ICD-10-PCS |
| DDY8CZZ | Intraoperative Radiation Therapy (IORT) of Anus | ICD-10-PCS |
| DDY8FZZ | Plaque Radiation of Anus | ICD-10-PCS |
| DF0 | Radiation Therapy, Hepatobiliary System and Pancreas, Beam Radiation | ICD-10-PCS |
| DF1 | Radiation Therapy, Hepatobiliary System and Pancreas, Brachytherapy | ICD-10-PCS |
| DF2 | Radiation Therapy, Hepatobiliary System and Pancreas, Stereotactic Radiosurgery | ICD-10-PCS |
| DFY07ZZ | Contact Radiation of Liver | ICD-10-PCS |
| DFY0CZZ | Intraoperative Radiation Therapy (IORT) of Liver | ICD-10-PCS |
| DFY0FZZ | Plaque Radiation of Liver | ICD-10-PCS |
| DFY17ZZ | Contact Radiation of Gallbladder | ICD-10-PCS |
| DFY1CZZ | Intraoperative Radiation Therapy (IORT) of Gallbladder | ICD-10-PCS |
| DFY1FZZ | Plaque Radiation of Gallbladder | ICD-10-PCS |
| DFY27ZZ | Contact Radiation of Bile Ducts | ICD-10-PCS |
| DFY2CZZ | Intraoperative Radiation Therapy (IORT) of Bile Ducts | ICD-10-PCS |
| DFY2FZZ | Plaque Radiation of Bile Ducts | ICD-10-PCS |
| DFY37ZZ | Contact Radiation of Pancreas | ICD-10-PCS |
| DFY3CZZ | Intraoperative Radiation Therapy (IORT) of Pancreas | ICD-10-PCS |
| DFY3FZZ | Plaque Radiation of Pancreas | ICD-10-PCS |
| DG0 | Radiation Therapy, Endocrine System, Beam Radiation | ICD-10-PCS |
| DG1 | Radiation Therapy, Endocrine System, Brachytherapy | ICD-10-PCS |
| DG2 | Radiation Therapy, Endocrine System, Stereotactic Radiosurgery | ICD-10-PCS |
| DGY07ZZ | Contact Radiation of Pituitary Gland | ICD-10-PCS |
| DGY0FZZ | Plaque Radiation of Pituitary Gland | ICD-10-PCS |
| DGY17ZZ | Contact Radiation of Pineal Body | ICD-10-PCS |
| DGY1FZZ | Plaque Radiation of Pineal Body | ICD-10-PCS |
| DGY27ZZ | Contact Radiation of Adrenal Glands | ICD-10-PCS |
| DGY2FZZ | Plaque Radiation of Adrenal Glands | ICD-10-PCS |
| DGY47ZZ | Contact Radiation of Parathyroid Glands | ICD-10-PCS |
| DGY4FZZ | Plaque Radiation of Parathyroid Glands | ICD-10-PCS |
| DGY57ZZ | Contact Radiation of Thyroid | ICD-10-PCS |
| DGY5FZZ | Plaque Radiation of Thyroid | ICD-10-PCS |
| DH0 | Radiation Therapy, Skin, Beam Radiation | ICD-10-PCS |
| DHY27ZZ | Contact Radiation of Face Skin | ICD-10-PCS |
| DHY2FZZ | Plaque Radiation of Face Skin | ICD-10-PCS |
| DHY37ZZ | Contact Radiation of Neck Skin | ICD-10-PCS |
| DHY3FZZ | Plaque Radiation of Neck Skin | ICD-10-PCS |
| DHY47ZZ | Contact Radiation of Arm Skin | ICD-10-PCS |
| DHY4FZZ | Plaque Radiation of Arm Skin | ICD-10-PCS |
| DHY5FZZ | Plaque Radiation of Hand Skin | ICD-10-PCS |
| DHY67ZZ | Contact Radiation of Chest Skin | ICD-10-PCS |
| DHY6FZZ | Plaque Radiation of Chest Skin | ICD-10-PCS |
| DHY77ZZ | Contact Radiation of Back Skin | ICD-10-PCS |
| DHY7FZZ | Plaque Radiation of Back Skin | ICD-10-PCS |
| DHY87ZZ | Contact Radiation of Abdomen Skin | ICD-10-PCS |
| DHY8FZZ | Plaque Radiation of Abdomen Skin | ICD-10-PCS |
| DHY97ZZ | Contact Radiation of Buttock Skin | ICD-10-PCS |
| DHY9FZZ | Plaque Radiation of Buttock Skin | ICD-10-PCS |
| DHYB7ZZ | Contact Radiation of Leg Skin | ICD-10-PCS |
| DHYBFZZ | Plaque Radiation of Leg Skin | ICD-10-PCS |
| DHYCFZZ | Plaque Radiation of Foot Skin | ICD-10-PCS |
| DM0 | Radiation Therapy, Breast, Beam Radiation | ICD-10-PCS |
| DM1 | Radiation Therapy, Breast, Brachytherapy | ICD-10-PCS |
| DM2 | Radiation Therapy, Breast, Stereotactic Radiosurgery | ICD-10-PCS |
| DMY07ZZ | Contact Radiation of Left Breast | ICD-10-PCS |
| DMY0FZZ | Plaque Radiation of Left Breast | ICD-10-PCS |
| DMY17ZZ | Contact Radiation of Right Breast | ICD-10-PCS |
| DMY1FZZ | Plaque Radiation of Right Breast | ICD-10-PCS |
| DP0 | Radiation Therapy, Musculoskeletal System, Beam Radiation | ICD-10-PCS |
| DPY07ZZ | Contact Radiation of Skull | ICD-10-PCS |
| DPY0FZZ | Plaque Radiation of Skull | ICD-10-PCS |
| DPY27ZZ | Contact Radiation of Maxilla | ICD-10-PCS |
| DPY2FZZ | Plaque Radiation of Maxilla | ICD-10-PCS |
| DPY37ZZ | Contact Radiation of Mandible | ICD-10-PCS |
| DPY3FZZ | Plaque Radiation of Mandible | ICD-10-PCS |
| DPY47ZZ | Contact Radiation of Sternum | ICD-10-PCS |
| DPY4FZZ | Plaque Radiation of Sternum | ICD-10-PCS |
| DPY57ZZ | Contact Radiation of Rib(s) | ICD-10-PCS |
| DPY5FZZ | Plaque Radiation of Rib(s) | ICD-10-PCS |
| DPY67ZZ | Contact Radiation of Humerus | ICD-10-PCS |
| DPY6FZZ | Plaque Radiation of Humerus | ICD-10-PCS |
| DPY77ZZ | Contact Radiation of Radius/Ulna | ICD-10-PCS |
| DPY7FZZ | Plaque Radiation of Radius/Ulna | ICD-10-PCS |
| DPY87ZZ | Contact Radiation of Pelvic Bones | ICD-10-PCS |
| DPY8FZZ | Plaque Radiation of Pelvic Bones | ICD-10-PCS |
| DPY97ZZ | Contact Radiation of Femur | ICD-10-PCS |
| DPY9FZZ | Plaque Radiation of Femur | ICD-10-PCS |
| DPYB7ZZ | Contact Radiation of Tibia/Fibula | ICD-10-PCS |
| DPYBFZZ | Plaque Radiation of Tibia/Fibula | ICD-10-PCS |
| DPYC7ZZ | Contact Radiation of Other Bone | ICD-10-PCS |
| DPYCFZZ | Plaque Radiation of Other Bone | ICD-10-PCS |
| DT0 | Radiation Therapy, Urinary System, Beam Radiation | ICD-10-PCS |
| DT1 | Radiation Therapy, Urinary System, Brachytherapy | ICD-10-PCS |
| DT2 | Radiation Therapy, Urinary System, Stereotactic Radiosurgery | ICD-10-PCS |
| DTY07ZZ | Contact Radiation of Kidney | ICD-10-PCS |
| DTY0CZZ | Intraoperative Radiation Therapy (IORT) of Kidney | ICD-10-PCS |
| DTY0FZZ | Plaque Radiation of Kidney | ICD-10-PCS |
| DTY17ZZ | Contact Radiation of Ureter | ICD-10-PCS |
| DTY1CZZ | Intraoperative Radiation Therapy (IORT) of Ureter | ICD-10-PCS |
| DTY1FZZ | Plaque Radiation of Ureter | ICD-10-PCS |
| DTY27ZZ | Contact Radiation of Bladder | ICD-10-PCS |
| DTY2CZZ | Intraoperative Radiation Therapy (IORT) of Bladder | ICD-10-PCS |
| DTY2FZZ | Plaque Radiation of Bladder | ICD-10-PCS |
| DTY37ZZ | Contact Radiation of Urethra | ICD-10-PCS |
| DTY3CZZ | Intraoperative Radiation Therapy (IORT) of Urethra | ICD-10-PCS |
| DTY3FZZ | Plaque Radiation of Urethra | ICD-10-PCS |
| DU0 | Radiation Therapy, Female Reproductive System, Beam Radiation | ICD-10-PCS |
| DU1 | Radiation Therapy, Female Reproductive System, Brachytherapy | ICD-10-PCS |
| DU2 | Radiation Therapy, Female Reproductive System, Stereotactic Radiosurgery | ICD-10-PCS |
| DUY07ZZ | Contact Radiation of Ovary | ICD-10-PCS |
| DUY0CZZ | Intraoperative Radiation Therapy (IORT) of Ovary | ICD-10-PCS |
| DUY0FZZ | Plaque Radiation of Ovary | ICD-10-PCS |
| DUY17ZZ | Contact Radiation of Cervix | ICD-10-PCS |
| DUY1CZZ | Intraoperative Radiation Therapy (IORT) of Cervix | ICD-10-PCS |
| DUY1FZZ | Plaque Radiation of Cervix | ICD-10-PCS |
| DUY27ZZ | Contact Radiation of Uterus | ICD-10-PCS |
| DUY2CZZ | Intraoperative Radiation Therapy (IORT) of Uterus | ICD-10-PCS |
| DUY2FZZ | Plaque Radiation of Uterus | ICD-10-PCS |
| DV0 | Radiation Therapy, Male Reproductive System, Beam Radiation | ICD-10-PCS |
| DV1 | Radiation Therapy, Male Reproductive System, Brachytherapy | ICD-10-PCS |
| DV2 | Radiation Therapy, Male Reproductive System, Stereotactic Radiosurgery | ICD-10-PCS |
| DVY07ZZ | Contact Radiation of Prostate | ICD-10-PCS |
| DVY0CZZ | Intraoperative Radiation Therapy (IORT) of Prostate | ICD-10-PCS |
| DVY0FZZ | Plaque Radiation of Prostate | ICD-10-PCS |
| DVY17ZZ | Contact Radiation of Testis | ICD-10-PCS |
| DVY1FZZ | Plaque Radiation of Testis | ICD-10-PCS |
| DW0 | Radiation Therapy, Anatomical Regions, Beam Radiation | ICD-10-PCS |
| DW1 | Radiation Therapy, Anatomical Regions, Brachytherapy | ICD-10-PCS |
| DW2 | Radiation Therapy, Anatomical Regions, Stereotactic Radiosurgery | ICD-10-PCS |
| DWY17ZZ | Contact Radiation of Head and Neck | ICD-10-PCS |
| DWY1FZZ | Plaque Radiation of Head and Neck | ICD-10-PCS |
| DWY27ZZ | Contact Radiation of Chest | ICD-10-PCS |
| DWY2FZZ | Plaque Radiation of Chest | ICD-10-PCS |
| DWY37ZZ | Contact Radiation of Abdomen | ICD-10-PCS |
| DWY3FZZ | Plaque Radiation of Abdomen | ICD-10-PCS |
| DWY47ZZ | Contact Radiation of Hemibody | ICD-10-PCS |
| DWY4FZZ | Plaque Radiation of Hemibody | ICD-10-PCS |
| DWY57ZZ | Contact Radiation of Whole Body | ICD-10-PCS |
| DWY5FZZ | Plaque Radiation of Whole Body | ICD-10-PCS |
| DWY5GDZ | Isotope Administration to Whole Body using Iodine 131 (I-131) | ICD-10-PCS |
| DWY5GFZ | Isotope Administration to Whole Body using Phosphorus 32 (P-32) | ICD-10-PCS |
| DWY5GGZ | Isotope Administration to Whole Body using Strontium 89 (Sr-89) | ICD-10-PCS |
| DWY5GHZ | Isotope Administration to Whole Body using Strontium 90 (Sr-90) | ICD-10-PCS |
| DWY5GYZ | Isotope Administration to Whole Body using Other Isotope | ICD-10-PCS |
| DWY67ZZ | Contact Radiation of Pelvic Region | ICD-10-PCS |
| DWY6FZZ | Plaque Radiation of Pelvic Region | ICD-10-PCS |

CPT = Common Procedural Terminology; HCPCS = Healthcare Common Procedure Coding System; ICD-9-PCS/ICD-10-PCS = International Classification of Disease, Ninth/Tenth Revision, Procedural Classification System.
